# Evolution of Antibiotic Tolerance Shapes Resistance Development in Chronic Pseudomonas aeruginosa Infections

**DOI:** 10.1128/mBio.03482-20

**Published:** 2021-02-09

**Authors:** Isabella Santi, Pablo Manfredi, Enea Maffei, Adrian Egli, Urs Jenal

**Affiliations:** a Biozentrum, University of Basel, Basel, Switzerland; b Division of Clinical Bacteriology and Mycology, University Hospital Basel, Basel, Switzerland; c Applied Microbiology Research, Department of Biomedicine, University of Basel, Basel, Switzerland; Institut Pasteur

**Keywords:** antibiotics, drug resistance evolution, tolerance

## Abstract

The widespread use of antibiotics promotes the evolution and dissemination of resistance and tolerance mechanisms. To assess the relevance of tolerance and its implications for resistance development, we used *in vitro* evolution and analyzed the inpatient microevolution of Pseudomonas aeruginosa, an important human pathogen causing acute and chronic infections. We show that the development of tolerance precedes and promotes the acquisition of resistance *in vitro*, and we present evidence that similar processes shape antibiotic exposure in human patients. Our data suggest that during chronic infections, P. aeruginosa first acquires moderate drug tolerance before following distinct evolutionary trajectories that lead to high-level multidrug tolerance or to antibiotic resistance. Our studies propose that the development of antibiotic tolerance predisposes bacteria for the acquisition of resistance at early stages of infection and that both mechanisms independently promote bacterial survival during antibiotic treatment at later stages of chronic infections.

## Abstract (precis)

Over the past decades, pan-resistant strains of major bacterial pathogens have emerged and have rendered clinically available antibiotics ineffective, putting at risk many of the major achievements of modern medicine, including surgery, cancer therapy, and organ transplantation. A thorough understanding of processes leading to the development of antibiotic resistance in human patients is thus urgently needed.

## INTRODUCTION

The great therapeutic achievements of antibiotics have been dramatically undercut by the steady evolution of survival strategies allowing bacteria to overcome antibiotic action (1, 2). Although resistance plays a major role in antibiotic-treatment failure, bacteria can use resilience mechanisms, such as tolerance, to survive antibiotic treatment (3). Whereas experts and the public are well aware of problems related to increasing resistance, pathogen tolerance is not common knowledge, despite being responsible for substantial morbidity and mortality (4). Resistance is generally drug specific and can be enhanced genetically through mutations modifying the drug target or by the acquisition of accessory genetic components, such as efflux pumps or antibiotic-modifying enzymes (5). Such events lead to a decrease of the effective antimicrobial concentration and an increase of the MIC, which corresponds to the lowest drug concentration needed to prevent pathogen growth. In contrast, tolerance is a situation where a fraction of the population can phenotypically resist the action of the antibiotic, while the MIC does not change. Importantly, while resistance is easily detected in clinical microbiology laboratories, tolerance is generally not assessed by standard clinical tests. Unless specific assays, like those described later in this work, are performed, tolerant strains will be classified as susceptible. Consequently, tolerance can lead to persistent infections despite a seemingly efficient treatment. In this case, a residual fraction of pathogens can resume growth after treatment is stopped, leading to infection relapses. For example, tolerance alters the kinetics of antibiotic killing without affecting MICs, leading to the prolonged treatment necessary for pathogen eradication. Tolerance is thus measured by the minimum duration of killing of a specific fraction of the population (6). This phenotype has been related to non- or slow-growing bacteria that are able to survive bactericidal antibiotics for extended times (7). Tolerance can be adopted by all cells of a bacterial culture or by only a subpopulation, called persisters (8). Bacterial populations with fractions of persisters are characterized by biphasic killing during treatment with bactericidal agents, where an initial rapid killing phase is followed by a phase of reduced killing (7).

Recent studies have shown that bacteria can rapidly evolve tolerance and persistence when exposed to antibiotics *in vitro* (9–12), suggesting that both represent successful strategies for bacteria to survive antibiotic treatment. In line with this, treatment efficacy during chronic infections was shown to be lost progressively without significant resistance development (13, 14). Although challenging to diagnose (15), tolerant variants exist among environmental bacteria (16) and clinical isolates of human pathogens (17–19). Recently, it was proposed that antibiotic tolerance facilitates the evolution of drug resistance under laboratory conditions (11, 20). However, it is still unclear if antibiotic tolerance plays a role in persistent infections and treatment failure (14, 19, 21) and if tolerance can facilitate resistance development in human patients (10, 15, 20).

Cystic fibrosis (CF) is the most common life-limiting, autosomal, recessively inherited disease in Caucasian populations, with the primary cause of death being respiratory failure resulting from chronic pulmonary infection (22). CF patients have reduced lung clearance capacity, leading to the development of lifelong chronic infections caused by opportunistic bacterial pathogens, such as Pseudomonas aeruginosa. Over time, treatment efficacy gradually declines, and increasing inflammatory damage leads to a fatal outcome (23). While infections are generally initiated by non-host-adapted strains found in the environment (24–26), P. aeruginosa undergoes significant microevolution during chronic infections of CF patient lungs (13, 27, 28). Despite recurrent application of high doses of antibiotics, a significant fraction of clinical isolates remains drug sensitive (13, 14). This argues that resistance development may not fully explain the long-term survival of pathogens in the lung and that other strategies, such as antibiotic tolerance, contribute to the highly persistent nature of such infections.

Here, we show that a substantial fraction of P. aeruginosa isolates from CF patient lungs has retained drug susceptibility but has evolved various degrees of multidrug tolerance. We demonstrate that recurrent exposure to high concentrations of antibiotics leads to the rapid development of tolerance, which generally precedes and boosts resistance development in P. aeruginosa. We show that tolerant strains display population heterogeneity with slow-growing subpopulations and that tolerance-mediated fitness costs can lead to the rapid loss of this phenotype after populations have acquired high-level resistance. Based on phenotypic and genotypic analysis of CF isolates, we propose that tolerance and resistance are alternative strategies contributing to P. aeruginosa persistence during long-term chronic infections.

## RESULTS

### P. aeruginosa develops multidrug tolerance during chronic infections of CF patient lungs.

**FIG 1:**
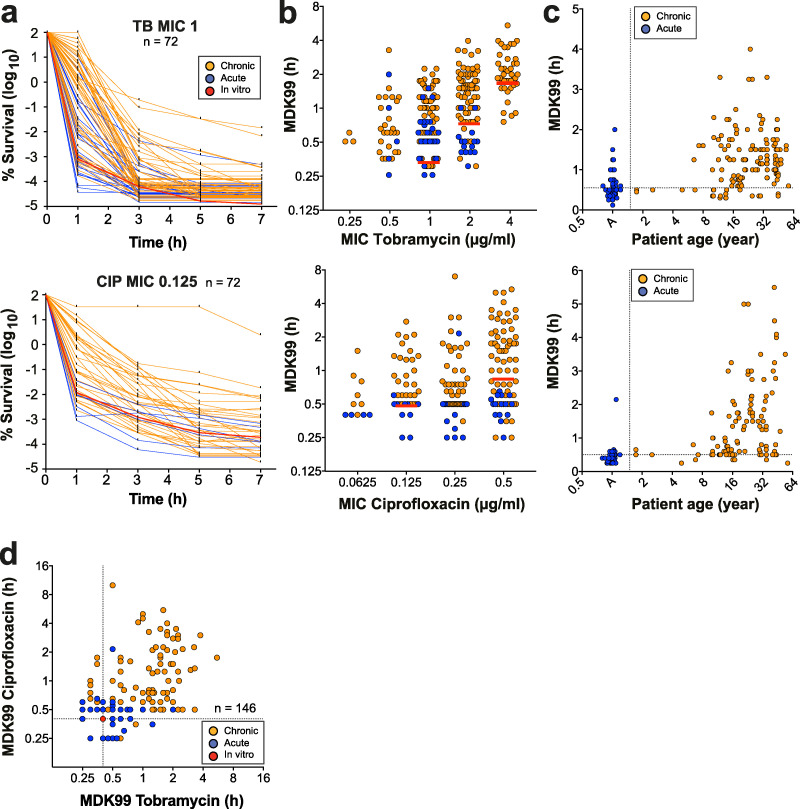
Selection of multidrug tolerance during chronic infections in CF patients. P. aeruginosa isolates from acute (blue, *n* = 30) or chronic (orange, *n* = 81) infections were tested for survival when challenged with 32 μg/ml tobramycin or 5 μg/ml ciprofloxacin. Only strains sensitive to the antibiotics of interest (according to EUCAST) were included in this analysis. (a) Killing kinetics of clinical isolates grouped according to their resistance levels (MICs). Survival rate is plotted over time. The red lines represent mean survival (± SD, *n* = 6) of the control lab strain PAO1. Data were normalized to the number of CFU at time zero. (b) MDK99 values are plotted against the MICs for the respective isolates. The MDK99 of P. aeruginosa laboratory strains with defined MICs are shown as red lines (mean ± SD). (c) MDK99 values are plotted against the age of CF patients at the time of strain isolation. Isolates from acute infections (A) are shown in the first column. (a to c) Upper and lower panels indicate tobramycin and ciprofloxacin treatment, respectively. (d) MDK99 values of P. aeruginosa CF patient isolates treated with tobramycin and ciprofloxacin. Only isolates that were sensitive to both tobramycin (MIC < 4) and ciprofloxacin (MIC < 1) were considered (*n* =146). The survival of the P. aeruginosa lab strain PAO1 is indicated by a red circle. (a to d) The data are from 2 independent experiments.

To explore the role of antibiotic tolerance in patients, we analyzed a large set of P. aeruginosa isolates (*n* = 539), including strains from chronically infected CF patients (*n* = 472), isolates from acute infections (*n* = 58 strains from 58 patients), and non-host-adapted control strains of laboratory or environmental origins (*n* = 9). CF patient isolates were sequentially isolated from 91 patients who were from 5 to 61 years old. For each strain, we analyzed antibiotic resistance profiles (MICs) as well as the ability to survive exposure to tobramycin and ciprofloxacin over time (Fig. 1a; see also Fig. S1a and S1b in the supplemental material). We chose these antibiotics because they have different modes of action and because they are used by clinicians to treat CF patients (29). Determination of MIC breakpoints according to clinical standards (30) revealed that clinically resistant strains were more common among isolates from chronic situations (22% tobramycin, 30% ciprofloxacin) than among isolates from acute infections (6% tobramycin, 17% ciprofloxacin). When analyzing the survival of isolates for which MIC values were below the clinical breakpoint (30) during exposure to high concentrations of tobramycin or ciprofloxacin, we observed large differences in killing kinetics within groups of isolates with identical levels of resistance (Fig. 1a; Fig. S1a and S1b). In particular, most isolates from chronic infections showed strongly enhanced tolerance, whereas isolates from acute infections were rapidly killed upon drug exposure (Fig. 1a and b; Fig. S1a to S1d). Minimum durations for killings of 99% (MDK99) and 99.99% (MDK99.99) were proposed as measures for tolerance and persistence, respectively (6). Because of the high survival rates of many clinical isolates, the MDK99.99 was not a useful measure for persistence. Instead, we decided to monitor the proportion of surviving bacteria after 1 h and after 7 h of treatment. While the former correlates well with other measures for tolerance, like the MDK99 (Fig. S1e), the latter value determines long-term survival without explicitly distinguishing between tolerance and persistence or a combination thereof. Importantly, most antibiotic-sensitive isolates from CF patient airways showed increased survival in the presence of both drugs (Fig. 1d), emphasizing the multidrug nature of tolerance.

10.1128/mBio.03482-20.1 FIG S1 Increased tolerance in isolates from patients with chronic lung infections. Susceptible P. aeruginosa isolates from patients with acute (blue, *n* = 58) or chronic (orange, *n*= 290) infections were tested for survival when challenged with 32 μg/ml of tobramycin or 5 μg/ml ciprofloxacin. Only strains sensitive to either tobramycin (c) or ciprofloxacin (d) were included in this analysis. (a and b) Killing kinetics of clinical isolates grouped according to their resistance levels (MIC) and treated with tobramycin (a) or ciprofloxacin (b). Survival rates are plotted over time. Red lines represent mean survival values (± SDs, *n* = 6) for the P. aeruginosa lab strain PAO1 and resistant variants with different MICs. Data were normalized to the number of CFU at time zero. (c, d) Killing curves of P. aeruginosa isolates treated with tobramycin (c) or ciprofloxacin (d) were binned based on the decay of survival. The average MIC, MDK99, and sample age (± SD) are shown for each cluster. (e) Survival of individual isolates after 1 h of killing with tobramycin (32 μg/m) or ciprofloxacin (5 μg/ml), plotted against MDK99. Download FIG S1, PDF file, 0.4 MB. Copyright © 2021 Santi et al. 2021 Santi et al. This content is distributed under the terms of the Creative Commons Attribution 4.0 International license.

Cluster analysis of the killing profiles delineated four different categories that were highly similar for both drug classes (Fig. S1b and S1c). Isolates from acute infections and control strains generally clustered in the first group, including the most rapidly killed isolates and isolates with the lowest levels of persisters. Also, CF isolates found in this group were from patients of the lowest age class (Fig. S1c and S1d). In contrast, most isolates from chronic CF infections grouped in clusters 2 to 4, which are characterized by minor changes in MICs but largely increased rates of survival after 1 and 7 h. Tolerance against tobramycin or ciprofloxacin was particularly pronounced in isolates from older patients (Fig. 1c).

From these data, we concluded that the ability to survive antibiotic exposure is widespread in patient isolates and that increased tolerance may evolve during chronic infections, allowing pathogens to survive a broad spectrum of antibiotics during chemotherapy (17, 18). The observed large differences in drug tolerance at any discrete level of antibiotic resistance reiterates that resistance and tolerance, although contributing to the same resilience phenomenon, are distinct phenotypes.

### Sequential treatment with a single antibiotic selects for tolerance and resistance.

**FIG 2:**
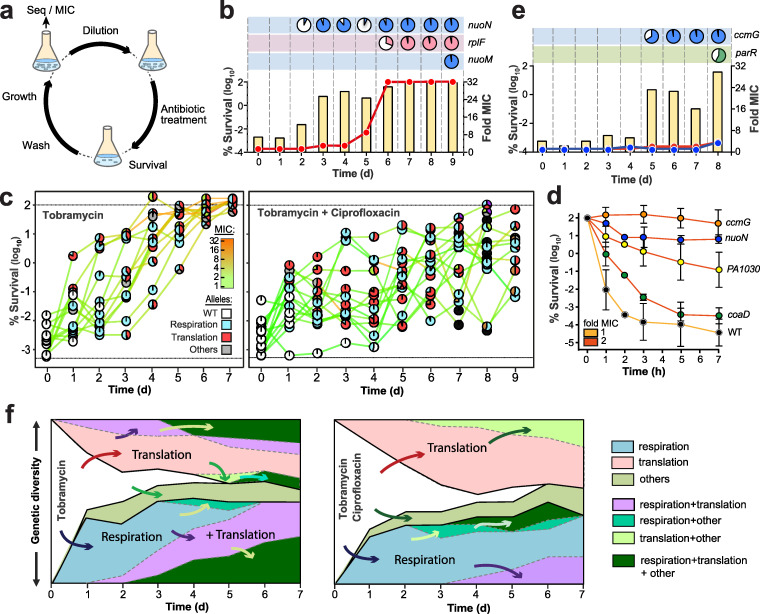
Evolution of antibiotic tolerance in P. aeruginosa. (a) Experimental design for iterative exposure of P. aeruginosa to bactericidal antibiotics (for details, see Materials and Methods). Seq, sequence. (b) Example of the P. aeruginosa response to cyclic exposure to tobramycin. The fraction of surviving cells (yellow bars) and MIC population values are indicated for tobramycin (red). Mutations acquired during selection are indicated in the boxes above the graph (see also Table S3 and Fig. S2 and S5 in the supplemental material), with pie charts representing population distribution of mutations (filled areas). d, days. (c) Evolution of the survival of lineages treated with tobramycin (left) or with tobramycin-ciprofloxacin (right). Survival is plotted together with MIC values for tobramycin (colored lines). MIC values for ciprofloxacin can be found in Fig. S4. Acquired mutations are indicated by pie charts, with white circles indicating wild-type (WT) genomes and the colors of invading alleles indicating specific functional classification. The lower dotted black line represents the detection limit for survival. (d) Increased survival to tobramycin of selected tolerance mutants. Individual alleles were crossed back from evolved lineages (Fig. S2) into the ancestral strain, and the survival of purified mutants was scored over time during treatment with tobramycin (32 μg/ml). MICs are indicated by colored lines. Values are means ± SDs from at least 3 experiments. (e) Example of P. aeruginosa response to cyclic exposure of tobramycin and ciprofloxacin. Details are as indicated in panel b, with MIC values of evolving populations shown in red (tobramycin) and blue (ciprofloxacin), respectively. (f) Evolutionary trajectories observed during selection with tobramycin (left panel) or with tobramycin and ciprofloxacin (right panel). The vertical axis indicates the proportion of lineages harboring mutations in genes of the indicated functional categories (genetic diversity). Independent evolutionary routes are separated by thicker black lines, with successive mutational events being indicated by arrows. Functional categories of mutated genes are indicated on the right.

The above data raised the question of which selective conditions favor the evolution of tolerance over resistance development in P. aeruginosa. To explore the selective regimens leading to the evolution of antibiotic tolerance and resistance, P. aeruginosa cultures were treated at daily intervals with high levels of tobramycin (32 μg/ml = 32× MIC), concentrations that are readily observed in the sputum of CF patients during aerosolized drug treatment (31). Daily cycles included resuspension of parallel overnight cultures in fresh medium containing tobramycin and drug exposure for 3 h, followed by washing and overnight growth in drug-free fresh medium (Fig. 2a). Bacterial survival was analyzed by plating after each killing phase, while MIC measurements and genome sequencing were performed each day after regrowth. Whereas less than 0.0001% of cells survived at the beginning of the experiment, all lineages rapidly adapted to the antibiotic regimen with a stepwise increase in survival rate. On average, tobramycin had lost its efficacy after 7 to 8 cycles, when all 13 independently evolved lineages had reached 10- to 32-fold-higher MICs than the ancestor (Fig. 2b and c; Fig. S2).

10.1128/mBio.03482-20.2 FIG S2 Mutations arising during single-drug selection as identified by whole-genome sequencing. Mutations in genes involved in respiration, protein synthesis, global regulators, DNA replication, or other functions are indicated in blue, red, green, orange, and gray, respectively. Circles indicate that a specific mutation was detected, and filled areas in the pie charts indicate mutation distribution in each lineage. For marked lineages (*), single colonies were randomly picked and allele combinations were analyzed by PCR. Specific mutations (underlined) were crossed back into the ancestral background and analyzed for their contribution to increased survival and MICs. MIC values and survival rates are reported for each day. Download FIG S2, PDF file, 1.0 MB. Copyright © 2021 Santi et al. 2021 Santi et al. This content is distributed under the terms of the Creative Commons Attribution 4.0 International license.

Time-resolved genome sequencing uncovered the appearance of specific mutant alleles during tobramycin selection. Allele linkage was inferred from quantification of sequencing coverage of the respective alleles and was experimentally confirmed by PCR and targeted sequencing (Fig. 2c; Fig. S2; Table S1). The majority of the genetic changes were nonsynonymous single nucleotide polymorphisms (SNPs) (Table S1), implying a functional role of the respective proteins in antibiotic survival. Most mutations mapped to genes of few functional categories, including respiration and energy metabolism (e.g., *nuoN*, *nuoM*, *ccoN1*, and *ccmF*), protein synthesis (e.g., *fusA* and *rplF*), and global regulation (e.g., *parRS* and *pmrAB*) (Fig. S2; Table S1). While some of these genes have been implicated in antibiotic tolerance (10), most of them are linked to target-specific (32) or indirect (33) resistance mechanisms. In line with this, survival rates and MIC values increased in parallel in most lineages (Fig. S2). From this and from the observation that, throughout most of the selection, MICs remained significantly below the concentration of tobramycin used in the experiment, we concluded that intermediate resistance levels boost population survival even at peak antibiotic concentrations. To test this, we isolated spontaneous resistance mutants of P. aeruginosa able to grow on agar plates with defined concentrations of tobramycin. Mutants for which MICs were 1, 2, 4, and 8 μg/ml indeed showed gradually increasing survival during treatment with 32 μg/ml tobramycin (Fig. S3a). These findings underscore that low-level resistance below the clinical resistance breakpoint can strongly promote survival at high therapeutic doses and emphasize that antibiotic tolerance should always be assessed in a resistance-neutral context, i.e., by comparing strains for which MICs are similar (Fig. 1a and b). In addition, resistance- and tolerance-mediated survival can be distinguished based on drug specificity and concentration range. Generally, tolerance, but not resistance, is a multidrug phenotype and is independent of the drug concentrations used.

10.1128/mBio.03482-20.3 FIG S3 P. aeruginosa adaptation to tobramycin. (a) Lineages evolved in the presence of tobramycin (see Fig. S2 in the supplemental material) were tested for tobramycin-mediated killing (32 μg/ml). Survival after 3 h of treatment is plotted against MIC values of the evolved population (*n *= 90). The time of isolation (days) of the evolved strains is indicated by the gray scale. The survival of nonadapted lab strains for which MICs are different (*n *= 12) is indicated by red dots (means ± SDs) (Fig. S1a). The dotted lines mark the breakpoints of resistance according to EUCAST (1): S, sensitive, and R, resistant. (b) Contributions of individual mutant alleles to survival during antibiotic treatment. Tolerance alleles from strains isolated during selection (Fig. S2) were crossed back into the ancestor and corresponding mutants treated with tobramycin (16 μg/ml). Survival is shown as means ± SDs from at least three experiments. Line coloring indicates MIC. Data in panels a and b were normalized to numbers of CFU before treatment. (c) Contribution of individual mutant alleles to survival during ciprofloxacin treatment. Selected alleles from evolved strains (Fig. S2) were crossed back into the ancestral strain, and the corresponding mutants were treated with ciprofloxacin (2.5 μg/ml). Line coloring represents MIC values of individual strains. Values are means ± SDs from at least three experiments. (d) Evolved lineages were analyzed by time-lapse microscopy for growth. The fraction of cells with extended lag phases (white circles) or reduced growth rates (black circle) are shown for each day of the selection. (e) The acquisition of a *nuoM* mutation in the *nuoN* hyper-tolerant strain background results in a loss of tolerance. Survival after tobramycin (32 μg/ml) administration is scored over time for the strains indicated. Values are means ± SDs from at least 3 experiments. Data were normalized to the number of CFU at 0 h. Download FIG S3, PDF file, 0.3 MB. Copyright © 2021 Santi et al. 2021 Santi et al. This content is distributed under the terms of the Creative Commons Attribution 4.0 International license.

10.1128/mBio.03482-20.4 FIG S4 Mutations arising during combination drug selection as identified by whole-genome sequencing. Lineage-specific mutations are indicated (Table S3). Mutations in genes involved in respiration, protein synthesis, global regulators, or other functions are indicated in blue, red, green, and gray, respectively. Circles indicate specific mutations, with filled areas in the pie charts indicating mutation distribution. For marked lineages (*), single colonies were randomly picked and allele combinations were analyzed by PCR. Specific mutations (underlined) were crossed back into the ancestral background and analyzed for their contribution to increased survival and MICs. MIC values and survival rates are reported for each day. Download FIG S4, PDF file, 0.7 MB. Copyright © 2021 Santi et al. 2021 Santi et al. This content is distributed under the terms of the Creative Commons Attribution 4.0 International license.

10.1128/mBio.03482-20.5 FIG S5 P. aeruginosa adaptation to double-drug treatment. (a) Tolerance levels and MICs of lineages evolved during selection with tobramycin (64× MIC, left panel) or ciprofloxacin (80× MIC, right panel). Strains generated by crossing back selected alleles (Table S3) into the ancestor are indicated in color. The average survival of the ancestor (*n* = 13) or lab strains, with corresponding MICs below the clinical breakpoint of resistance (1) (dotted lines), is indicated by red lines. The dotted lines mark the breakpoints of resistance according to EUCAST (EUCAST, Breakpoint tables for interpretation of MICs and zone diameters, version 8.1, 2018): S, sensitive; R, resistant. (b) Example of tolerance evolution in lineage 19. Samples from sequential time points (gray scale) were treated with tobramycin (64 μg/ml), and survival was measured over time. MICs of the evolved populations are indicated by the colored lines. Dotted line, minimal duration of killing for 99% of the population (MDK99). (c) Time-lapse analysis of lineages evolved in the presence of tobramycin and ciprofloxacin reveals cells with different growth behaviors. The fraction of cells of individual lineages with a prolonged lag phase (white circles) or with a reduced growth rate (black circle) are shown as a function of time. (d) Evolved lineages show cross tolerance against polymyxin B. Lineages that evolved during single- or double-drug selection were analyzed for polymyxin B resistance (MIC) and for survival during 3 h of treatment with 15 μg/ml polymyxin B. The time of isolation (days) of individual strains during the selection window is indicated in gray shading. Colors indicate the presence of SNPs in genes known to confer polymyxin resistance. Red line, average survival of the ancestor (*n *= 14). Data were normalized to the number of CFU at 0 h. (e) Contribution of individual mutant alleles to antibiotic survival. Selected alleles from evolved strains (Fig. S2 and S4) were crossed into the PAO1 ancestor, and MIC values (right) and the survival (left) of the resulting mutants were determined. Survival was determined after 3 h of treatment with tobramycin at 16 μg/ml (*x* axis) or ciprofloxacin at 2.5 μg/ml (*y* axis). Survival scores of the ancestor are indicated by a red circle. Values are means ± SDs (*n* > 3). Download FIG S5, PDF file, 0.3 MB. Copyright © 2021 Santi et al. 2021 Santi et al. This content is distributed under the terms of the Creative Commons Attribution 4.0 International license.

10.1128/mBio.03482-20.9 TABLE S1 List of mutations acquired during the evolution with single- or double-drug treatment. Download Table S1, XLSX file, 0.03 MB. Copyright © 2021 Santi et al. 2021 Santi et al. This content is distributed under the terms of the Creative Commons Attribution 4.0 International license.

Genome sequencing revealed the sequential invasion and fixation of specific alleles during daily treatment intervals (Fig. 2b and c), with SNPs in genes involved in respiration and energy metabolism generally preceding the acquisition of resistance mutations (Fig. S2). To test their role in survival, a selection of the respiration-related alleles was crossed back into the ancestral background. While some alleles showed moderate effects (e.g., *nuoD* and *ccmF*) (Fig. S3b), a point mutation in *nuoN* (G300D) encoding a subunit of respiratory complex I strongly increased survival during tobramycin (Fig. 2d) and during ciprofloxacin (Fig. S3c) treatment. The *nuoN* mutant showed an extended lag phase (on average, 7 h long) when exiting from stationary phase, a phenomenon typically observed in hyper-tolerant strains (Fig. S3d) (9). Mutations in *nuoN* also confer drug tolerance in Escherichia coli (10). Remarkably, the tolerance phenotype of this lineage was lost by a second-site mutation in *nuoM* after penetration of a mutation in *rplF* conferring high-level resistance to tobramycin (Fig. S2, lineage 1; Fig. S3e). Thus, tolerance mutations can impose fitness costs, which can be compensated for through second-site mutations after populations have reached high levels of resistance. It is possible that tolerant variants transiently invade pathogen populations during antibiotic therapy, explaining the difficulties of gauging the actual contribution of tolerance to resistance development and treatment failures.

In sum, these results indicated that, in contrast to what had been reported for E. coli (20), treatment with tobramycin alone drives parallel evolution of tolerance and resistance in P. aeruginosa. This difference is likely due to different antibiotics being used in these experiments and the respective differences in the spontaneous resistance rates.

### Sequential treatment with a drug combination strongly selects for tolerance.

We next challenged P. aeruginosa with a combination of tobramycin and ciprofloxacin at therapeutic doses. Survival again rapidly increased in multiple independent lineages, with killing efficiencies dropping 4 to 5 orders of magnitude after 7 to 9 cycles. Unlike with single-drug treatment, all lineages evolved high levels of tolerance against both tobramycin and ciprofloxacin without developing significant levels of resistance (Fig. 2c and e; Fig. S4, S5a, and S5b; Table S1). Tolerant lineages often developed subpopulations with an extended lag phase or slower growth (Fig. S5c). In contrast to most lineages obtained during single-drug treatment, lineages obtained during double-drug treatment exhibited lag phases (on average, 3 h) that precisely matched the duration of drug exposure, indicating evolutionary trajectories minimizing fitness costs under these conditions. Similar precise lag-phase extensions were also observed in E. coli upon exposure during defined treatment windows (9). Several of the tolerant lineages showed cross-tolerance against membrane-active compounds like polymyxin B (Fig. S5d), and this phenomenon was also observed in lineages evolved with tobramycin alone. Thus, treatment with a combination of bactericidal antibiotics strongly favors the development of multidrug-tolerant P. aeruginosa (2). The observation that some of the tolerant lineages acquired intermediate-level resistance against both drugs at later time points (Fig. S4, lineages 16, 17, 20, 23, and 25) indicated that during combination therapy, tolerance-based survival may promote the development of multidrug resistance (11, 34).

Time-resolved population sequencing and PCR-based SNP confirmation of individual clonal members showed that sublineages coexisted for several selection cycles and generally conferred a large increase in survival (Fig. 2e; Fig. S4; Table S1). Crossing back specific alleles into the ancestral lineage revealed their multidrug tolerance phenotype, as indicated by large increases of survival (Fig. 2d; Fig. S3c, S5a, and S5b). Most mutations that increased tolerance against both drugs led to a small increase in resistance to tobramycin (MIC, 2 μg/ml^−1^) but not to ciprofloxacin, an effect that cannot account *per se* for the large increase in tolerance (see the MIC controls in Fig. S3a and S5a). Also, several tolerant strains showed reduced MICs of ciprofloxacin (see the supplemental data and Fig. S5e), suggesting that under these conditions, tolerance development is strongly favored. As in the single-drug regimen, tolerance alleles mapped to genes involved in respiratory and energy metabolism, including respiratory complex I, cytochrome *c* maturation, and the cytochrome *c* oxidase complex (Fig. 2c and d; Table S1). In line with this, altered respiration has recently been linked to increased persister levels in E. coli (35). Finally, SNPs in genes coding for a toxin-antitoxin system (*PA1029*-*PA1030*) and cell metabolism (*coaD*) were associated with increased tolerance. *PA1030* encodes a toxin of the RES domain family that was recently shown to interfere with NAD metabolism (36–38).

Typically, tolerant mutants evolved directly from a susceptible ancestor and were able to outcompete other coemerging linages, irrespective of differences in MICs (Fig. 2c; Fig. S4, lineages 13, 19, and 22). Once established, such lineages provided the genetic background for the fixation of additional mutations that either conferred moderate but clinically relevant resistance (e.g., *gyrA* [39] in lineage 20, *rplF* [40] or *fusA* in lineage 22, *parR* [33] in lineage 25) or further increased tolerance (e.g., *nuoN* in lineage 15 or *hxcR* in lineage 19) (Fig. S4). Alleles conferring substantial resistance against one drug could invade transiently but were not stably maintained under these conditions (e.g., lineages 14 and 17). Several SNPs mapped to *fusA1*, the gene for elongation factor G (EF-G) (Fig. S4 and S5e; Table S1), a protein that was recently associated with both tolerance and aminoglycoside resistance *in vivo* (32, 41, 42). Most *fusA1* alleles conferred only moderate increases in survival against both tobramycin and ciprofloxacin and were often outcompeted by other sublineages (Fig. S4 and S5e).

In sum, these experiments revealed that although selection with one or two antibiotics yielded different mutations, similar genes and functional pathways were affected under both conditions. In general, the acquisition of mutations in components of the translation apparatus was preceded by mutations in genes involved in the respiratory process or vice versa (Fig. 2e). These results suggested that these two pathways synergistically influence antimicrobial sensitivity and survival, possibly via their central role in the generation and consumption of energy.

### Respiratory mutations generate slow-growing subpopulations.

**FIG 3:**
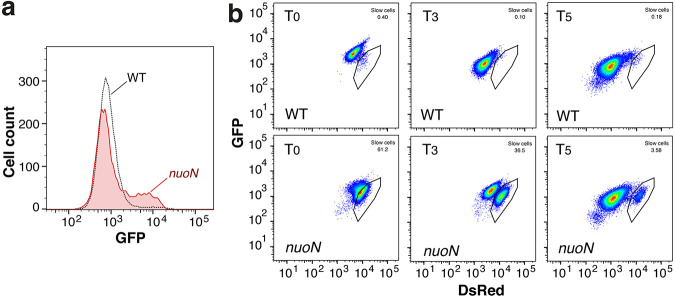
The *nuoN* hyper-tolerant mutant strain shows a subpopulation with low membrane potential and reduced growth rates. (a) Flow cytometry analysis of overnight cultures of the PAO1 wild type and isogenic *nuoN G300D* mutant incubated with DiBAC_4_, an indicator dye of membrane potential that is taken up only by cells with collapsed membrane potential (67). GFP, green fluorescent protein. (b) Flow cytometry of the PAO1 wild type and isogenic *nuoN G300D* mutant containing a plasmid with constitutive expression of TIMER^bac^ (46). Bacteria were grown overnight, diluted into fresh medium, and analyzed by fluorescence-activated cell sorting (FACS) over time. Recordings are shown at time point 0 (T0) and after 3 and 5 h of incubation (T3 and T5, respectively). Black outlines indicate slow-growing subpopulations that were observed only in the tolerant mutant strains. DsRed, red fluorescent protein.

To better understand antibiotic tolerance, a selection of the mutants emerging from experimental evolution was characterized in more detail. For example, a Gly-to-Asp exchange in the NuoN transmembrane subunit of respiratory complex I (RCI) conferred high levels of tolerance and increased levels of persisters (Fig. 2d; Fig. S5e [see also the other supplemental data]). This Gly residue is positioned in the immediate vicinity of helix 7 of NuoN, a region known to be involved in proton-pumping activity (43). In line with this, we found that a subpopulation of *nuoN* mutants had reduced membrane potential (Fig. 3a). Because uptake of aminoglycosides depends on the membrane potential (44), this may explain tobramycin tolerance but not tolerance against ciprofloxacin, whose uptake is independent of the proton-motive force (45). Alternatively, cells with low membrane potential may have reduced growth rates or extended lag phases (Fig. S3d) during outgrowth in fresh medium. In line with this idea, we observed distinct subpopulations of slowly growing bacteria when analyzing the *nuoN* mutant expressing TIMER^bac^ (46) as an indicator for growth rates (Fig. 3b). Of note, mutations in *nuo* genes were found in several lineages of P. aeruginosa isolated from CF patients, indicating that these genes are under selection *in vivo* (47). Future work will need to correlate drug tolerance with slow growth and/or extended lag phases of these mutant strains at the single-cell level.

### Tolerance facilitates resistance development in P. aeruginosa.

**FIG 4:**
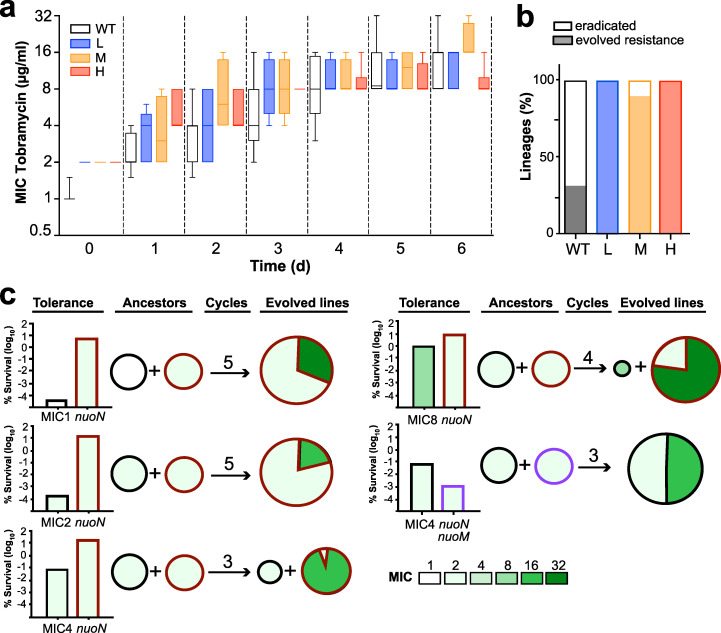
Tolerance facilitates antibiotic resistance development. (a) Tolerance does not accelerate resistance development. Resistance (MIC) evolved over time in strains with different initial levels of tolerance (H, high [*nuoN_G300D_*]; M, medium [PA1030*]; L, low [*nuoD_R551P_*]) (Fig. S5) by serial daily exposure to tobramycin (32 μg/ml). Box plots are shown with quartiles and standard deviations from at least 3 experiments. (b) Tolerance facilitates the evolution of resistance lineages. Strains with different levels of tolerance as in panel a were analyzed during daily exposure to tobramycin. The fraction of independent lineages that were eradicated (white) or developed resistance (black) during drug treatment are indicated (*n* = 6). (c) Tolerance provides a strong advantage during development of antibiotic resistance. One-to-one mixtures of low (black)- and high (red)-tolerance P. aeruginosa strains were challenged daily with tobramycin (32 μg/ml). Tolerance levels of the initial strains are indicated as survival after 3 h of drug treatment (left). Daily treatment intervals were as described in the legend of Fig. 2a, with the number of selection cycles indicated for each experiment. Genetic changes were determined by whole-population genome sequencing at the end of the selection. Resistance levels and resistance allele distributions are indicated. Red and black frames indicate resistance evolution in the high- and low-tolerance ancestors, with initial MIC levels indicated.

The above results indicated that tolerance evolves rapidly when P. aeruginosa is periodically exposed to high concentrations of antibiotics and that tolerance development often precedes resistance. To test if tolerance influences the rate of resistance development, we periodically exposed the P. aeruginosa wild type and mutants with distinct levels of tolerance (low, medium, high) to tobramycin (Fig. S6a and S6b). While all strains gradually evolved resistance over time, the rates of resistance acquisition were similar in all strains (Fig. 4a; Fig. S6a; Table S1). However, tolerant lineages were more likely to survive the initial selection than the nontolerant PAO1 lab strain (Fig. 4b), indicating that tolerance provides bacterial populations with a significant advantage to evolve resistance. To test this, we performed antibiotic selection experiments with a 1:1 mixture of the PAO1 wild type and isogenic high-tolerance variants (Fig. 4c). We found that PAO1 was consistently outcompeted by its isogenic high-tolerance counterpart when mixtures were repeatedly exposed to high concentrations of tobramycin. Resistance was systematically acquired by strains exhibiting the highest level of tolerance, even when they had coevolved with nontolerant strains with higher initial MIC levels. Even in the few rare cases where the original low-tolerance competitor strain was still detected at the end of the treatment regimen, mutations conferring resistance were acquired by the high-tolerance ancestor (Fig. 4c). Importantly, while the hyper-tolerant *nuoN* mutant was highly successful in acquiring resistance mutations, its low-tolerance isogenic *nuoN nuoM* descendant (Fig. S3e) failed to acquire resistance (Fig. 4c).

10.1128/mBio.03482-20.6 FIG S6 (a) Mutant strains with different level of tolerance (wild type, low [nuoD_R551P_], medium [PA1030*], and high [nuoN_G300D_]) were treated with tobramycin (16 μg/ml), and survival was scored over time. Values are means ± SDs from at least three experiments. Data were normalized to the number of CFU at 0 h. (b) Experimental design for iterative exposure of mixtures of P. aeruginosa strains with low and high levels of tolerance to bactericidal antibiotics. Overnight cultures were diluted at a 1:1 ratio into fresh medium and challenged for 3 h with 32 μg/ml tobramycin. After antibiotic washout, cultures were resuspended in fresh medium and grown overnight. The cycle was repeated for several days until resistance increased. Whole-population genome sequencing was performed for the ancestor mixture and the evolved lineages. Download FIG S6, PDF file, 0.2 MB. Copyright © 2021 Santi et al. 2021 Santi et al. This content is distributed under the terms of the Creative Commons Attribution 4.0 International license.

From this, we concluded that tolerance provides P. aeruginosa with a strong selective advantage when challenged with bactericidal antibiotics by promoting survival and by increasing the probability of resistance development. Tolerance not only may increase the chance of resistance alleles to emerge and spread but also may increase the stringency of selection, as emerging resistant variants need to be able to outcompete higher numbers of persisting individuals during periods of unconstrained growth.

### Evolution of tolerance and resistance during chronic infections of P. aeruginosa.

**FIG 5:**
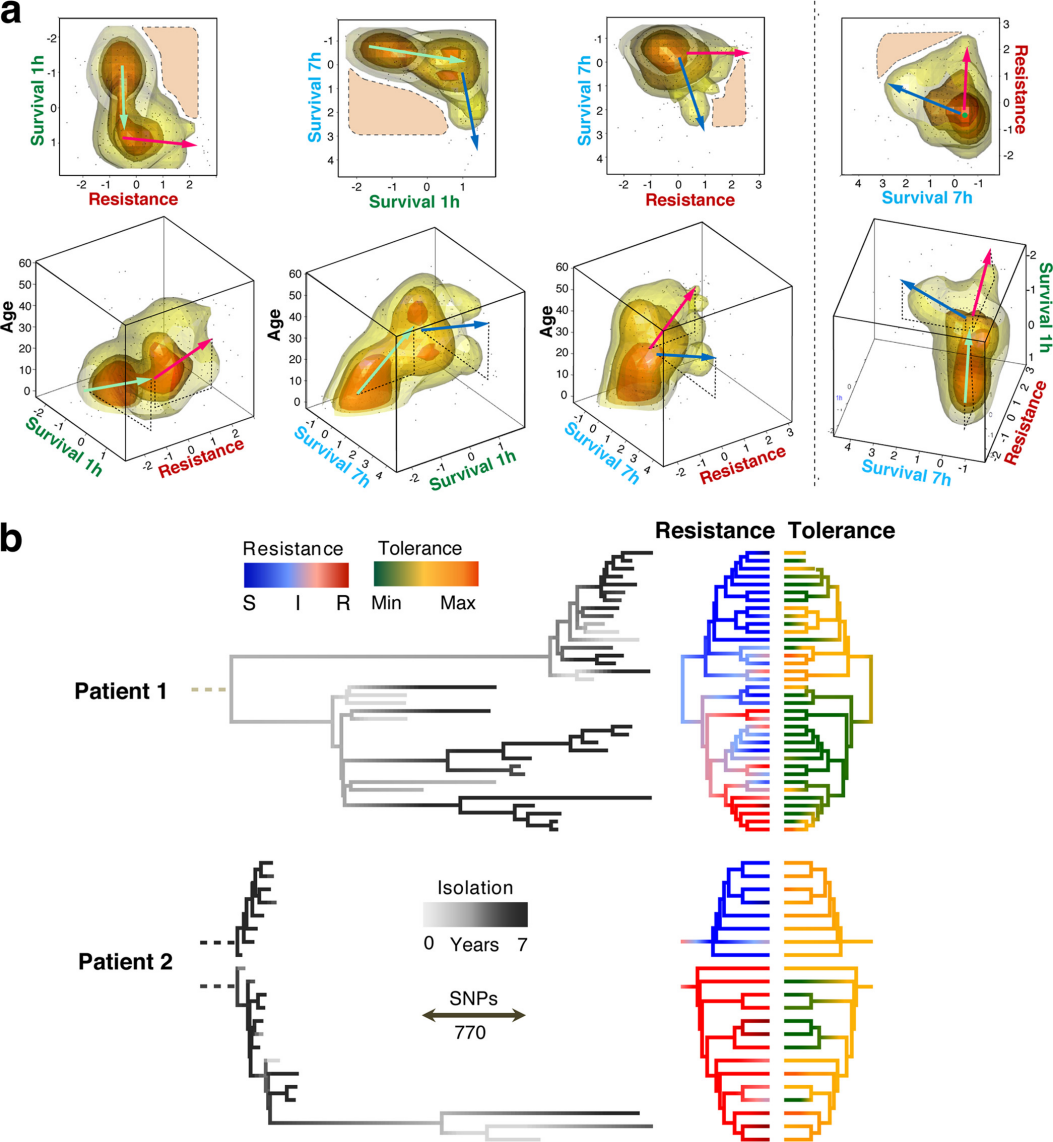
Microevolution of tolerance and resistance during chronic P. aeruginosa infections. (a) Illustration of antimicrobial phenotypes during host microevolution. Strains (dots) and their density distribution (clouds) are represented as a function of three antimicrobial parameters and patient age at the time of strain isolation: survival after 1 h, survival after 7 h, and resistance (MIC). P. aeruginosa isolates analyzed are from patients with acute (*n* = 67, age = zero) and chronic (*n* = 312, 91 patients who were from 5 to 61 years old) infections (Fig. S7). In order to eliminate resistance effects on tolerance, only sensitive strains were analyzed (tobramycin MIC < 4 μg/ml or ciprofloxacin MIC < 1 μg/ml [*n* = 496 comparisons]). Survival rates are shown as standardized log_10_ values. Resistance values correspond to log_2_(MICs). Cloud colors indicate data densities, with numbers increasing from yellow to orange. Arrows indicate phenotype progression, with color indicating different parameters (green, survival after 1 h; blue, survival after 7 h; red, resistance; black, age of the patient). Areas with dashed outlines delineate phenotype profiles that were rarely observed. (b) Genome-based phylogeny (85,579 variant positions) of P. aeruginosa strains from two CF patients. The branch lengths of the trees on the left indicate genetic differences (SNPs). Dashed roots represent the extrapolated clonal ancestors. Colors of the terminal branches indicate isolation time within a 5-year window (gray scale), resistance (sensitive [S], blue; intermediate [I], white; resistant [R], red), and tolerance (lowest tolerance, green; intermediate tolerance, yellow; highest tolerance, orange). Resistance is plotted as the average log_2_ fold variation from intermediate MICs of five antibiotics (Fig. S7). Tolerance is plotted as the average survival scores for antibiotics to which no resistance was observed.

To better understand the evolutionary trajectories leading to antibiotic resilience in human patients, we reconsidered the resistance and tolerance of 252 drug-sensitive isolates of P. aeruginosa derived from a cohort of 91 CF patients. To be able to assess resistance and tolerance in parallel, we analyzed only strains that had retained sensitivity to at least one of the two drugs used in this study. We determined resistance (MIC) as well as survival rates after 1 and 7 h of treatment for individual patient isolates or from patients sampled repeatedly over periods of up to 10 years. Visualizing these phenotypic distributions across patient age at the time of isolation illustrates the evolutionary trends of antibiotic resilience in the clinical data set. In order to avoid resistance effects in the assessment of tolerance, we reported resistance against antibiotic A versus tolerance against antibiotic B and vice versa (496 comparisons) (Fig. 5a).

10.1128/mBio.03482-20.7 FIG S7 Microevolution of survival and antibiotic resistance during chronic infections of CF patient lungs. Genome-based phylogeny (85,579 positions) of P. aeruginosa strains from two CF patients sampled over a period of 5 years. Branch lengths of trees (left) indicate genetic differences (substitutions). Dashed roots represent extrapolated clonal ancestors. The colors of the terminal branches indicate (from left to right) isolation time during longitudinal sampling, fitness (growth in LB medium), resistance (Res) (average log_2_ fold variation from the intermediate resistance MIC of each antibiotic), and survival (Surv) (for standardized survival scores for each antibiotic, see Materials and Methods). Survival is reported for all strains, including isolates for which the MIC of a given antibiotic is high. TM, tobramycin; CIP, ciprofloxacin; PolB, polymyxin B; MP, meropenem. Download FIG S7, PDF file, 0.5 MB. Copyright © 2021 Santi et al. 2021 Santi et al. This content is distributed under the terms of the Creative Commons Attribution 4.0 International license.

Strains isolated from younger CF patients generally displayed low resistance and low tolerance. With increasing age, the number of drug-tolerant isolates gradually rose, while resistance to tobramycin or ciprofloxacin remained low (Fig. 5a). Hyper-tolerant but susceptible (low-MIC) strains were often found in patients at an intermediary age (15 to 30 years old), while increased numbers of resistant isolates were observed only at higher patient ages (above 30 years old) and generally lagged behind tolerance development (Fig. 5a). Intriguingly, we observed two distinct pathogen subpopulations in older patients: isolates that were highly resistant to both antibiotics and isolates that had retained low-level resistance but had acquired a hyper-tolerant phenotype (Fig. 5a). These observations indicated that P. aeruginosa gradually adapts during chronic infections by first evolving increased antibiotic tolerance, followed by an increase of multidrug resistance or hyper-tolerance at later stages of the infection. Importantly, while moderate levels of resistance and tolerance coexisted in some isolates, strains displaying both high levels of resistance and tolerance were rarely observed (Fig. 5a). In fact, the distribution of tolerance and resistance in clinical isolates from late stages of infection as depicted in Fig. 5a (top right plot) suggested that both phenotypes independently contribute to the same selective phenomenon.

Next, we investigated resistance and tolerance phenotypes of P. aeruginosa isolates collected longitudinally from two CF patients over several years (Fig. 5b; Fig. S7). Whole-genome sequences of 58 isolates were compared to references from GenBank revealing highest similarities with strain RIVM-EMC2982, a clinical isolate from the Netherlands (GenBank accession no. CP016955.1), 12-4-4(59), a strain isolated from the blood of a burn patient (accession no. NZ_CP013696.1 [48]), and W36662, a strain isolated from a cancer patient (accession no. CP008870.2). All strains from patient 1 belong to a single clonal clade (W36662), while sequences of isolates of patient 2 indicated a recent and ongoing superinfection by different clones [RIVM-EMC2982 and, more recently, 12-4-4(59)]. In line with previous observations (13), parallel evolution of coexisting P. aeruginosa lineages was observed in both patients. As expected, longitudinal sampling did not directly reflect genetic evolution, as more recent isolates did not necessarily descend from previous isolates but rather emerged from a common ancestor (13). Phylogenetic analyses indicated that in patient 1, an early split of the clonal lineages separated populations into two clades with similar distributions during sampling over the next years (Fig. 5b). Given the significant evolutionary distance between these clades, we cannot distinguish whether they are the result of microevolution of a common ancestor or a coinfection by two closely related isolates.

In both patients, the highest levels of resistance against different classes of antibiotics (tobramycin, ciprofloxacin, and meropenem) were observed within the same phylogenetic branch, while the other lineages showed much lower levels of resistance but significant levels of tolerance (Fig. 5b). Isolates with low MICs but high tolerance showed rates of survival similar to those of resistant isolates, underscoring that tolerance is highly successful during chronic infections. In both patients, the genetic divergence (i.e., branch length) was significantly higher in the clades exhibiting high-level resistance, irrespective of isolation time (Fig. 5b), suggesting that different resilience phenotypes may result from different levels of genetic alterations. This is in line with the multidrug nature of tolerance (Fig. 1d), which may represent a simpler genetic solution during multidrug therapy than evolving multiple drug-specific resistance mechanisms. Importantly, multidrug-resistant strains that retained sensitivity to one specific drug (e.g., tobramycin) generally displayed high-level tolerance to this antibiotic (Fig. S7).

Although the sample density did not permit identifying genetic variations correlating with antibiotic tolerance or resistance by genome-wide association, sequential acquisition of known resistance alleles (*fusA1*, *pmrB*, *gyrA*, *gyrB*, *oprD*) (49) or mutations in genes that were shown in this study to enhance tolerance (*nuoG*, *nuoN*, *ccmG*, *PA1030*, *PA1549*) were readily observed in the clinical isolates (Fig. S8). Together, these phylogenetic analyses propose that different antimicrobial survival strategies (i.e., tolerance and resistance) evolve and coexist in chronically infected patients. Our data also argue that at later stages of chronic infections of CF airways, mutually exclusive evolutionary trajectories result in increased resistance and drug tolerance, respectively.

10.1128/mBio.03482-20.8 FIG S8 Alleles associated with antibiotic resistance and tolerance in P. aeruginosa isolates from CF patients. Genome-based phylogeny of P. aeruginosa strains from two CF patients as shown in Fig. 5b. The colors of the terminal branches of the phylogeny indicate the resistance score as described in Fig. 5b. Mutations involved in resistance to aminoglycosides (red), fluoroquinolones (blue), carbapenems (green), and other β-lactams (orange) are indicated for each P. aeruginosa isolate from patients 1 and 2. Mutations in genes that were shown to contribute to tolerance in this study are highlighted in gray. Download FIG S8, PDF file, 0.6 MB. Copyright © 2021 Santi et al. 2021 Santi et al. This content is distributed under the terms of the Creative Commons Attribution 4.0 International license.

## DISCUSSION

Based on our findings, we propose a dynamic evolutionary model of acquisition of tolerance and resistance during antibiotic chemotherapy. Typically, chronic infections are sparked by a naive nontolerant and sensitive strain. Despite early treatment with antibiotics, initial colonization of CF patient airways does not seem to result in resistance development in P. aeruginosa populations (50). Our data argue that antibiotic exposure selects for increased tolerance early during CF patient lung colonization, in particular during treatment with combinations of antibiotics administered jointly or serially (11, 51). Preferential development of tolerance over resistance may be due to a wide range of possible tolerance mechanisms, providing a larger target size for mutations alleviating antibiotic stress (9). Moreover, due to the multidrug-resistant phenotype of P. aeruginosa, tolerance development may be particularly favored during chemotherapy with combinations of drugs, a treatment strategy that is often used for CF patients by combining inhalation and systemic therapy (29). In agreement with this, we show here that recurrent exposure of P. aeruginosa to high doses of tobramycin and ciprofloxacin leads to the rapid evolution of hyper-tolerance but not high-level resistance. In contrast, resistance is generally drug specific, with mutations being commonly limited to direct drug targets. Once established, tolerance likely provides populations with a significant survival advantage, facilitating the acquisition of genetic changes that further increase antibiotic resilience (11, 20, 52). Our studies indicate that during chronic infections in CF lungs, this process ultimately leads to genetic changes conferring high-level resistance to multiple antibiotics or to further increments of tolerance levels (Fig. 5a and b). Independent evolutionary trajectories leading to different mechanisms of antibiotic resilience are in line with the observation that isolates with strongly diverging resistance levels are observed in individual patients, a phenomenon that may relate to physical separation and local microevolution in CF lungs (13, 14).

Although antimicrobial tolerance is underappreciated and still largely neglected clinically (4), we propose that it plays an important role during in-host microevolution, leading to rapid pathogen adaptation and the discharge of antibiotic stress. Our data imply that tolerance can not only precede and promote resistance development during early stages of infections but also serve as an alternative mechanism enabling long-term survival of pathogens during continued antibiotic exposure during chronic infections. The observation that some of the isolates from older CF patients had retained low MICs and low tolerance to different antibiotics argues for additional protective mechanisms, such as pathogen encapsulation into biofilm structures (53). Limited bacterial intermixing and different selective pressures in different lung regions may explain much of the differences in tolerance and resistance profiles of isolates from the same patient (13).

In addition to the fitness advantage conferred by antibiotic tolerance, low-level resistance below the clinical breakpoint of antibiotics may contribute to survival during chronic CF infections. Genetic studies indicated that the target size for mutations conferring low-level tobramycin resistance in P. aeruginosa is considerable (54). Moreover, mutations conferring low-level tobramycin or ciprofloxacin resistance also confer substantial fitness advantages at antibiotic concentrations in the sub-MIC range (55). In this study, we showed that low-level resistance also contributes to increased survival at very high antibiotic concentrations. Thus, mutations conferring low-level resistance show a tolerance phenotype at concentrations above the MIC. This argues that the contribution of resistance below the clinical breakpoint to pathogen survival should not be neglected, as it may well help explain clinical treatment failures (56). It is also possible that the “tolerance aspect” of low-level resistance may help explain its role in the development of high-level resistance (57). While we observed that mutations conferring tolerance often resulted in small increases of MICs, their effect on survival was disproportionally higher than bona fide resistance mutations with similar MIC values. Moreover, they generally showed a multidrug phenotype, while mutations causing resistance were generally drug specific. Thus, despite similarities in promoting survival during antibiotic treatment, tolerance and low-level resistance are clearly distinct phenomena and based on different molecular and cellular mechanisms.

Altogether, our findings shed new light on the forces shaping antibiotic resistance development in the human pathogen P. aeruginosa and emphasize the need for compounds with an improved capacity to eliminate tolerant cells (58). Our results may also help to optimize antibiotic treatment strategies during chronic infections to minimize the risk of resistance development. For example, because tolerance influences the rate of *de novo* emergence of single- and double-drug resistances, detailed knowledge about tolerance development during chronic infections should influence treatment decisions regarding mono-drug versus multidrug therapy (34). Access to such information will require the development of simple and rapid tools for routine monitoring of antibiotic tolerance alongside with resistance.

## MATERIALS AND METHODS

### Bacterial strains and culture conditions.

Strains used in this study are listed in Table S2 in the supplemental material. Unless otherwise stated, P. aeruginosa PAO1 and all E. coli strains were grown at 37°C in Luria-Bertani (LB) medium (59) with shaking at 170 rpm or, alternatively, statically and solidified with 1.3% agar when appropriate. For P. aeruginosa, tetracycline was used at 100 μg/ml (E. coli, 12.5 μg/ml) and Congo red dye was added to a final concentration of 0.04%.

10.1128/mBio.03482-20.10 TABLE S2 List of strains and plasmids used in this study. Download Table S2, DOCX file, 0.02 MB. Copyright © 2021 Santi et al. 2021 Santi et al. This content is distributed under the terms of the Creative Commons Attribution 4.0 International license.

10.1128/mBio.03482-20.11 TABLE S3 Oligonucleotides used in this study. Download Table S3, DOCX file, 0.01 MB. Copyright © 2021 Santi et al. 2021 Santi et al. This content is distributed under the terms of the Creative Commons Attribution 4.0 International license.

### Plasmids and oligonucleotides.

Plasmid and primers used in this study are listed in Tables S2 and S3.

### Molecular biology procedures.

Cloning was carried out in accordance with standard molecular biology techniques. Plasmids pEX18Tc-nuoN*, pEX18Tc-nuoD*, pEX18Tc-nuoM*, pEX18Tc-PA5221*, pEX18Tc-ccmG*, pEX18Tc-coaD*, pEX18Tc-parS*, pEX18Tc-*FusA*Y630C, pEX18Tc-*FusA*Q678L, and pEX18Tc-PA1549* were produced by ligation of nuoN* (primers A and B from lineage 14, day 7 genomic DNA [gDNA]), nuoD* (primers C and D from lineage 14, day 1 gDNA), nuoM* (primers E and F from lineage 14, day 9 gDNA), *fusA* Y630C (primers G and H from lineage 2, day 10 gDNA), *fusA* Q678L (primers G and H from lineage 7, day 4 gDNA), PA1549* (primers I and J from lineage 23, day 1 gDNA), ccmG* (primers K and L from lineage 13, day 7 gDNA), coaD* (primers M and N from lineage 13, day 5 gDNA), parS* (amplified with primers O and P from lineage 18, day 5 gDNA), and PA5221* (amplified with primers Q and R from lineage 17, day 2 gDNA) PCR fragments between the HindIII and XbaI sites of pEX18Tc (60). The strains carrying the mutations *fusA* T671A, *fusA* R680C, ccmF*, and PA1030* PA0686* are single-colony isolates from lineages 8 (day 2), 6 (day 4), 24 (day 2), and 7 (day 7), respectively.

### Evolution experiments.

Independent overnight cultures of a single PAO1 colony in 5 ml of LB medium were diluted into fresh LB medium to an optical density (OD) of 0.12 and challenged with antibiotics for 3 h at 37°C and 170 rpm in a flask. Aliquots of the cultures were sampled, diluted to the appropriate dilutions, and plated on LB medium plates. CFU were measured after overnight incubation and over a 2-day period to make sure that all CFU had appeared. After antibiotic treatment, cells were washed two times in LB medium to remove antibiotics and the pellet was inoculated in 5 ml of fresh LB medium for another cycle. Survival was scored by plating dilutions before and after treatment. In the evolution with a single drug, tobramycin was used at 32 μg/ml. In the evolution with two drugs, the combination of tobramycin and ciprofloxacin at 16 μg/ml and 2.5 μg/ml, respectively, was used. Every day before the drug treatment, (i) 1 ml of the overnight culture was pelleted and the pellet frozen at −80°C in 1 ml of LB medium–10% dimethyl sulfoxide (DMSO) for further analysis, (ii) 1 ml of the overnight culture was pelleted and the pellet frozen for genomic DNA extraction, and (iii) the MIC of the population was estimated (see below for details). For competition experiments, the followed protocol was the same except that the overnight cultures of the competing strains were diluted into 20 ml fresh LB medium to a final OD of 0.06 each and challenged with 32 μg/ml tobramycin for 3 h at 37°C and 170 rpm in a flask. In order to assess the right ratio (1:1) of the competing strains, at day 0, before the drug treatment, 1 ml of the mixture was pelleted and the pellet frozen for genomic DNA extraction.

### Antibiotic survival assays.

To measure survival under antibiotic treatments, overnight cultures, each grown from a single colony in LB medium in the case of the mutants or from 10% DMSO stock for the evolved lineages, were diluted to an OD of 0.12 into fresh LB medium supplemented with a fixed concentration of antibiotics in order to avoid possible changes in the modes of action of the different drugs used. At the time points indicated in the figures, aliquots of the cultures were sampled, diluted, and plated on LB medium plates. CFU were counted after overnight incubation and over a 2-day period to make sure that all CFU had appeared.

### MIC assays.

MICs were quantified based on adaptations of methods described elsewhere (61, 62). Briefly, an overnight culture was diluted in LB medium to an inoculum of 1 × 10^6^ CFU /ml, incubated in a range of 2-fold antibiotic dilutions, and grown for 16 to 20 h at 37°C with shaking. After incubation, the OD at 600 nm (OD_600_) was measured. We defined the MIC value as the lowest antibiotic concentration at which no growth was observed.

### Microscopy.

Bacteria were grown overnight (OD_600_, 2 to 3) in LB liquid medium, diluted 1:100 in fresh LB medium, and then transferred to a slide with a 1% agarose pad buffered with LB medium. Bacteria were imaged by time-lapse microscopy using a DeltaVision microscope (Applied Precision) equipped with a 100× oil immersion objective and an environmental chamber maintained at 35°C. Images were recorded on a phase-contrast microscope using a CoolSNAP HQ2 camera. Images were processed using SoftWoRx software (Applied Precision). “Lag phase” was defined as the time between the start of the movie and the initiation of cell elongation.

### Genomic DNA extraction, whole-genome sequencing, and coverage analyses.

For each evolved lineage, 1 ml of the overnight culture was daily pelleted and frozen at −80°C. Clinical isolates of P. aeruginosa were grown overnight in liquid LB medium, and pellets from 1 ml of culture were collected. Genomic DNA was then extracted using the GenElute bacterial genomic DNA kit (Sigma; NA2120-1KT). Genomic DNA integrity was monitored by 0.6% agar gel electrophoresis, and gDNA was sent to the Genomics Facility Basel for library preparation and Illumina sequencing (ETH Zurich Department of Biosystems Science and Engineering, Basel, Switzerland). Library preparation and sample barcoding were generated with the Nextera XT approach (Illumina), and library quality was checked with a fragment analyzer (Advanced Analytical). PE125 sequencing runs were performed on 95 libraries at a time in a single HiSeq lane (Illumina) with a targeted coverage of ca. 100×. Reads were mapped onto the genome of the reference strain, Pseudomonas aeruginosa PAO1 (GenBank accession no. NC_002516), with Bowtie 2 (63), and small polymorphisms and structural and coverage variants were spotted with SAMtools (64) and a collection of in-house Perl scripts. Only genetic variations that were observed in more than 10% of the reads at at least one time point are reported here. In many cases, the evolutionary context was simple enough to infer the presence of sublineages partially or fully genetically identifiable by coverage analysis of the population genome sequencing. For example, in lineage 1 (Fig. S2), one can see the subpopulation exhibiting an intergenic mutation between PA1554 and PA1555 invading the lineage over the first 5 days, while the number of wild-type variants drops. At day 6, the wild-type variant of the intergenic region becomes dominant again in the evolving population. At the same time and at similar levels, another variation in the *nuoN* gene quickly invaded the lineage, indicating that these two mutations represent two competing sublineages. Furthermore, often genetic fixations could be observed in many lineages when 100% of sequencing reads exhibited a stable mutation over several time points (e.g., lineages 2, 8 to 11, 13 to 19, 21 to 23, and 25). In these cases, we could establish sequential acquisition of genetic variations (when present in more than 10% of the population).

### Phylogeny of clinical strains.

Whole-genome sequences of 58 chronic-infection isolates from patients 1 and 2 (Table S4) were compared to references from GenBank, revealing highest similarities with strain RIVM-EMC2982, a clinical isolate from the Netherlands (GenBank accession no. CP016955.1), 12-4-4(59), a strain isolated from the blood of a burn patient (accession no. NZ_CP013696.1), and W36662, a strain isolated from a cancer patient (accession no. CP008870.2). Similarity to the references was assessed by the similarity BLAST score (in bits) of the largest contigs from each assembly. The phylogenetic tree of the chronic infection strains from patients 1 and 2 were based on the 85,579 chromosomal positions where polymorphism has been observed in at least one of the clinical strains sequenced. The evolutionary history was inferred in MEGA7 (65) by using the maximum likelihood method based on the Tamura-Nei model (66). Phenotypically color-coded trees were generated with the R package phytools (http://github.com/liamrevell/phytools). Differences between the clonal branches (i.e., clades closely related to the same reference) were between 17,400 and 27,400 bp at conserved sites (including polymorphisms and indels).

10.1128/mBio.03482-20.12 TABLE S4 Genome sequences of clinical isolates. Download Table S4, DOCX file, 0.02 MB. Copyright © 2021 Santi et al. 2021 Santi et al. This content is distributed under the terms of the Creative Commons Attribution 4.0 International license.

### Computation of tolerance and resistance scores, clustering, and cloud fitting.

For longitudinal samples isolated from single patients (Fig. 5b), resistance scores (*R*) were computed as the average of log_2_ fold variations from the intermediary clinical resistance level (*I*) for each drug considered (*d*): tobramycin (*I*, 6 μg ml^−1^), ciprofloxacin (*I*, 1 μg ml^−1^), polymyxin B (*I*, 4 μg ml^−1^), colistin (*I*, 4 μg ml^−1^), and meropenem (*I*, 6 μg ml^−1^). Therefore, for a given strain, considering five drugs,

(1) $$R=\frac{1}{\text{drugs}}\cdot \overset{\text{drugs}}{\underset{d}{\sum }}{\text{log}}_{2}\left(\frac{{\text{MIC}}_{d}}{I_{d}}\right)$$

Tolerance scores (*T*) correspond to the average of log_10_ values for a 3-h survival assay (SURV; see above) with different drugs (*d*) and were standardized per patient (Pat). Only survival values from nonresistant strains were considered to compute the latter tolerance score, with a MIC cutoff that minimized the impact of resistance traits on the survival assay: tobramycin (*I*, 2 μg ml^−1^), ciprofloxacin (*I*, 1 μg ml^−1^), and polymyxin B (*I*, 8 μg ml^−1^). Therefore, for a given strain considering one to three drugs,

(2) $$T=\frac{1}{\text{drugs}}\cdot \overset{\text{drugs}}{\underset{d\;}{\sum }}\left(\frac{{\text{SURV}}_{d}-\bar{{\text{SURV}}_{d|\text{Pat}}}}{\sigma {\text{SURV}}_{d|\text{Pat}}}\;\right)$$

For the analysis of tolerance and resistance over 317 isolates (Fig. 5a), the resistance scores correspond to the MIC of tobramycin or ciprofloxacin normalized to the clinically relevant resistance breakpoints, similarly to in equation 1. Tolerance scores correspond to log_10_ values for a 3-h survival assay (see above) with either tobramycin or ciprofloxacin standardized by drug over the whole data set, similarly to in equation 2 but with an average and standard deviation (SD) from the whole strain data set. In order to assess the resistance and tolerance of the same strains, we report only the tolerance score for strains that were sensitive to at least one of the two drugs, tobramycin (only strains for which the MIC was <4 μg ml^−1^) or ciprofloxacin (only strains for which the MIC was ≤1 μg ml^−1^), and we provide tolerance scores for drugs different from the one used to assess resistance. This made it possible to gauge the multidrug tolerance feature under at least one effective antimicrobial treatment. Clustering of killing profiles was performed with the “cluster” R library using the pam (partitioning around medoids) function. Appropriate numbers of clusters (i.e., 4) were selected by the “elbow method” on Ball and Hall sores computed with the intCriteria function from the clusterCrit R library. Cloud fitting of tolerance and resistance scores of strains over the age of patients at isolation time was performed with the sm.density function of the “Sm” R package. Naive isolates and acute-infection isolates were used to simulate the prime infecting strains and were assigned a patient age of zero. To avoid useless interpolations, regions with no or poor data coverage were excluded.

### Statistics.

Data acquired were analyzed using GraphPad Prism version 6.03 for Mac and R.3.5.0, with different packages as described in appropriate sections. Mean values and standard deviations were obtained from at least three independent experiments (biological replicates). All the documented results are highly reproducible. No statistical method was used to predetermine the sample size.

### Ethics statement.

The clinical P. aeruginosa isolates used in this study were cultured from patient samples collected for routine microbiological testing at the University Hospital, Basel, Switzerland. Subculturing and analysis of bacteria were performed anonymously. No additional procedures were carried out on patients. Cultures were sampled by following regular procedures with written informed consent, in agreement with the guidelines of the Ethikkommission beider Basel EKBB.

### Data availability.

All genetic variations observed during *in vitro* evolution of P. aeruginosa PAO1 are referenced in the Table S1. Raw sequencing data of clinical strains have been deposited in the NCBI Sequence Read Archive (SRA) under accession numbers available at Table S4.
