# Supplementary figures and images for: Evolution of Antibiotic Tolerance Shapes Resistance Development in Chronic Pseudomonas aeruginosa Infections
Source: mBio. 2021 Feb 9;12(1):e03482-20. doi: 10.1128/mBio.03482-20 (PMC7885114; doi:10.1128/mBio.03482-20)

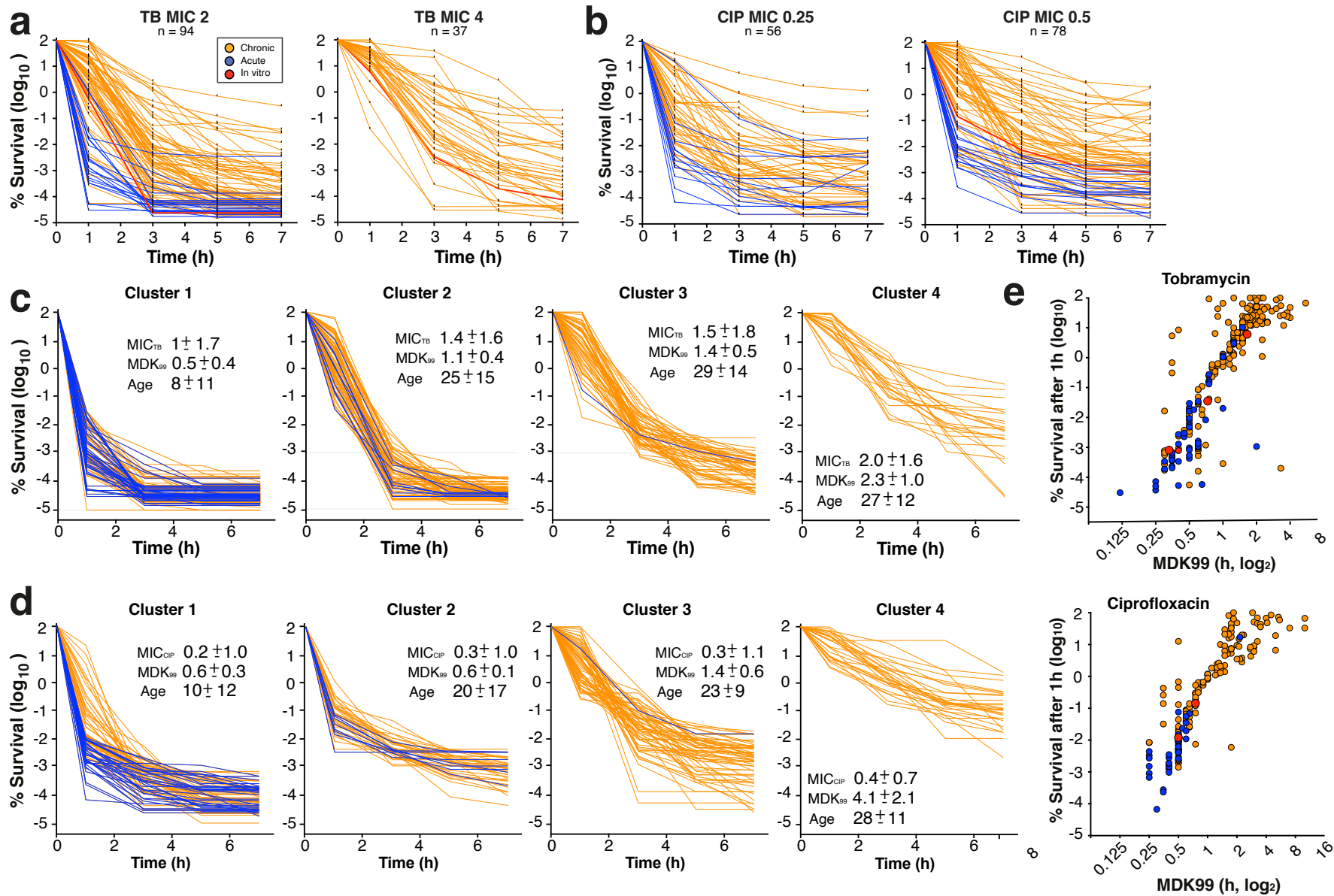

Supplement: FIG S1 [file mBio.03482-20-sf001.pdf]

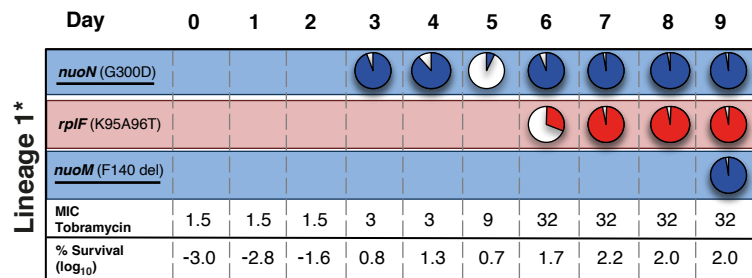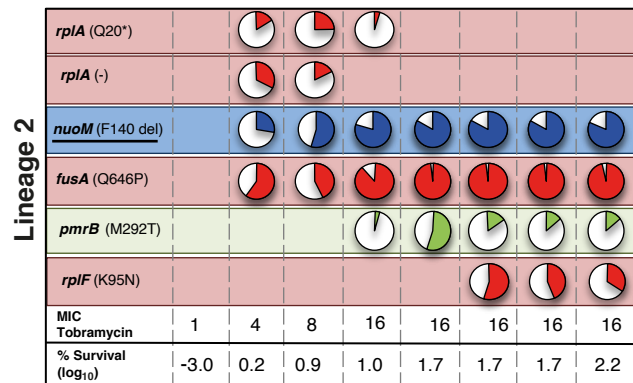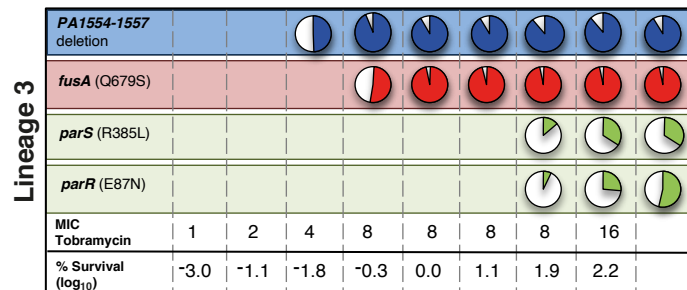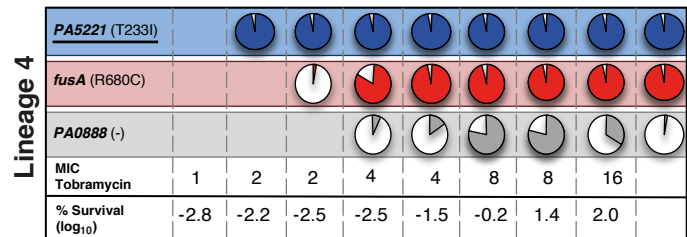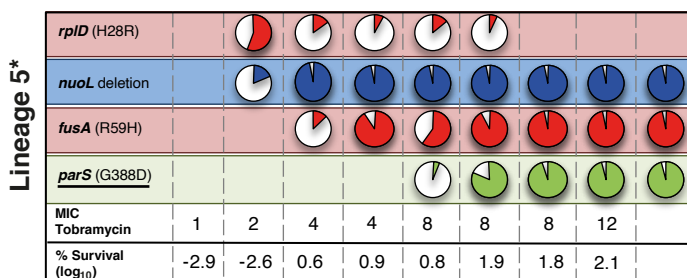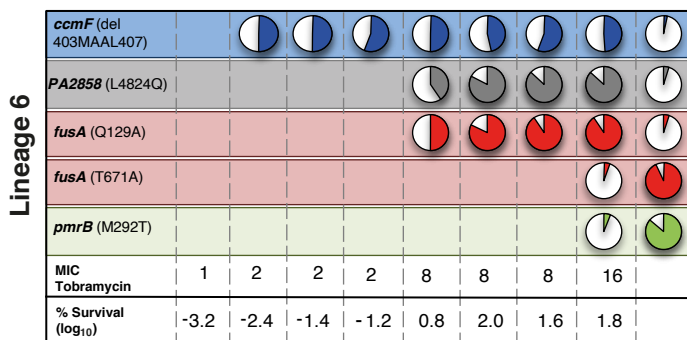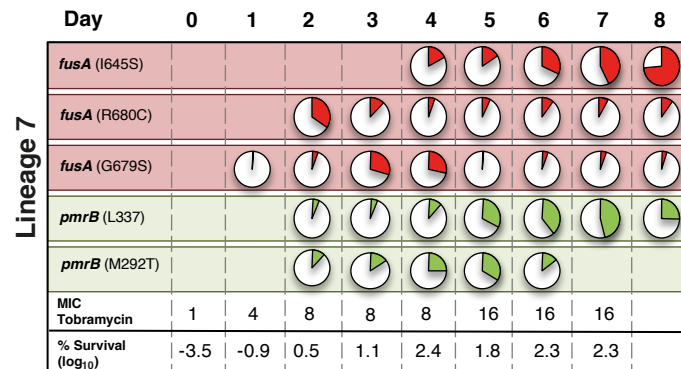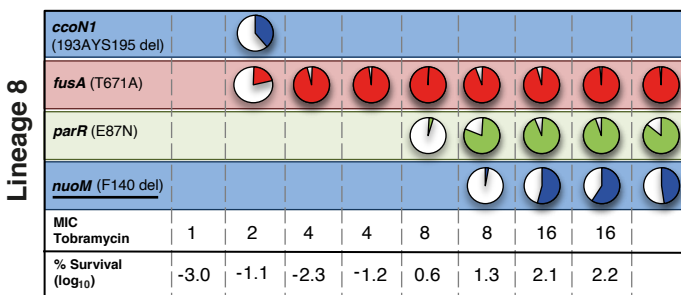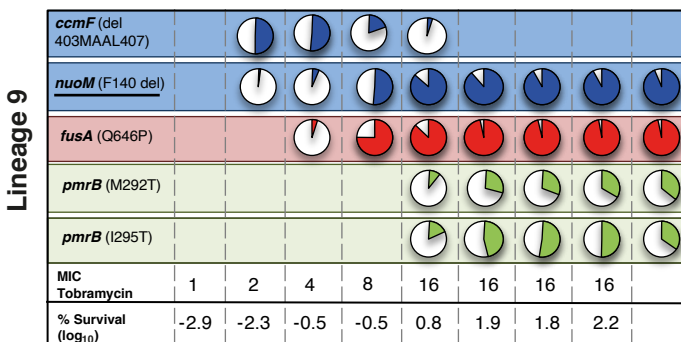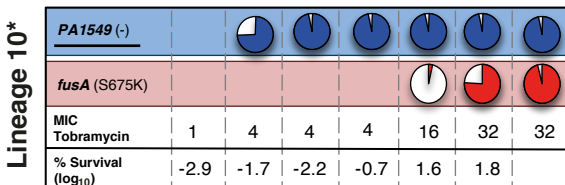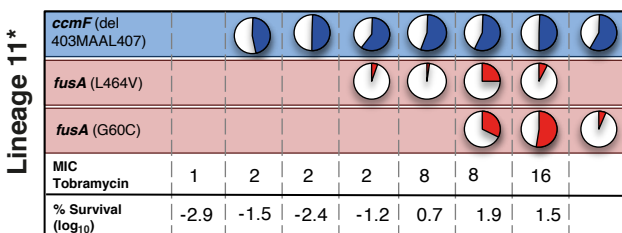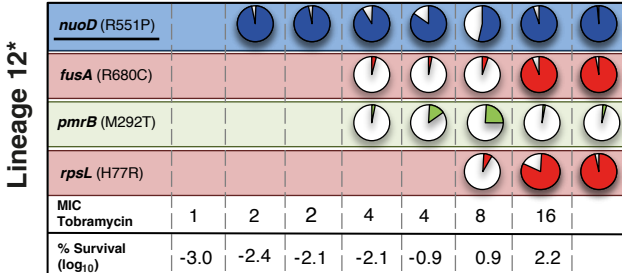

Supplement: FIG S2 [file mBio.03482-20-sf002.pdf]

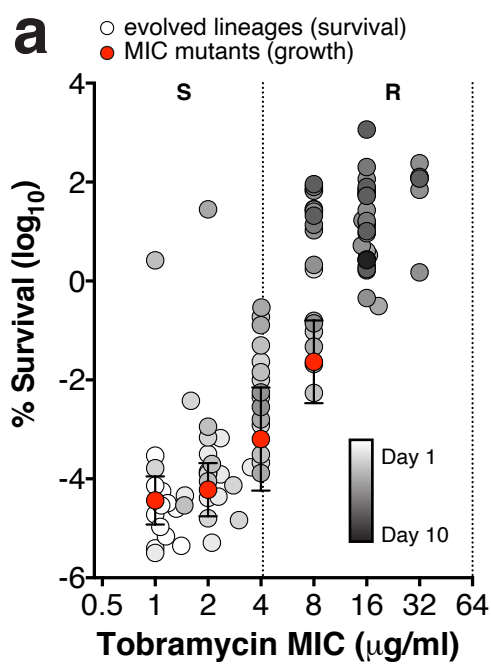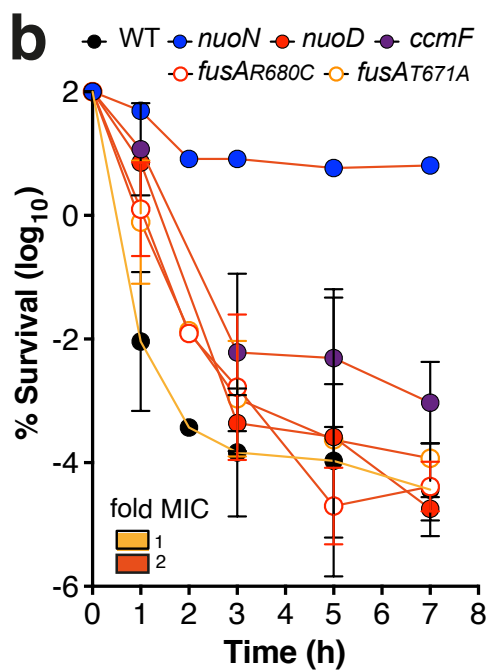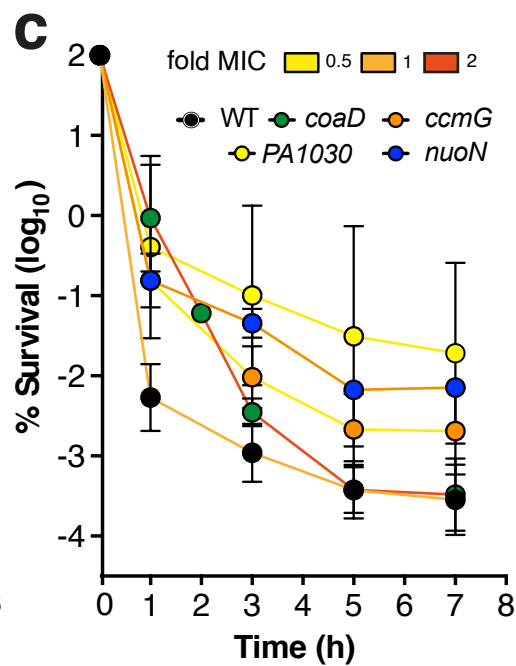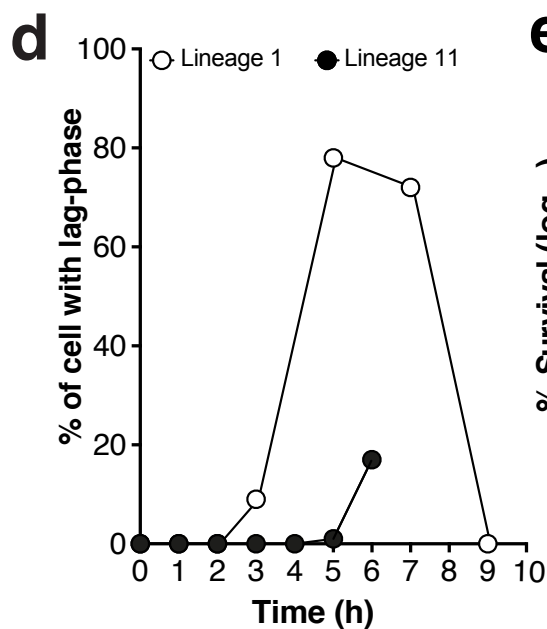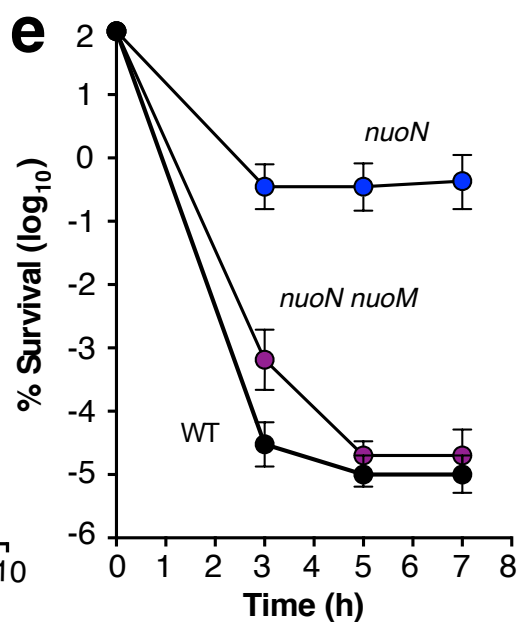

Supplement: FIG S3 [file mBio.03482-20-sf003.pdf]

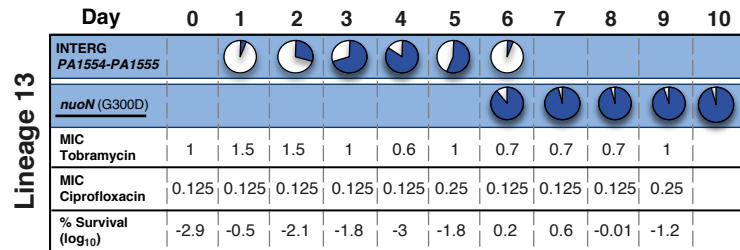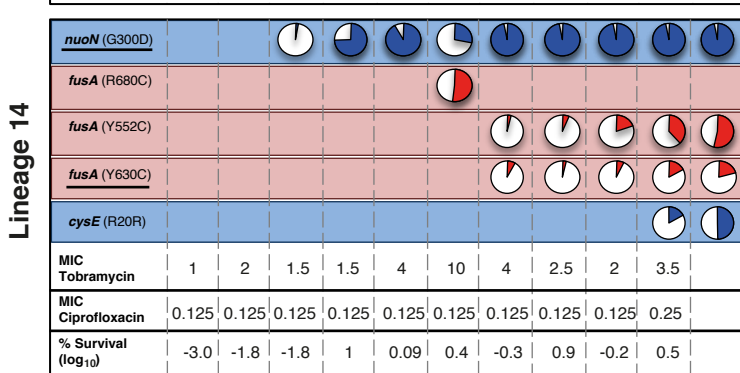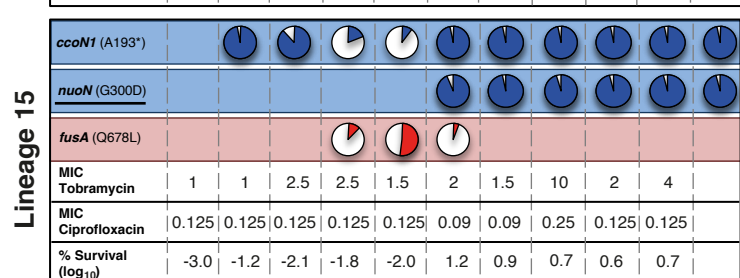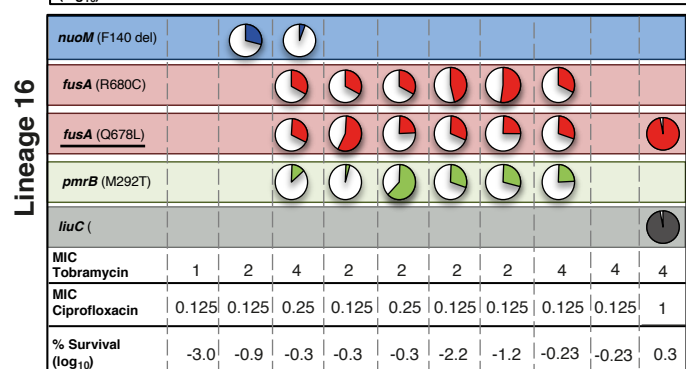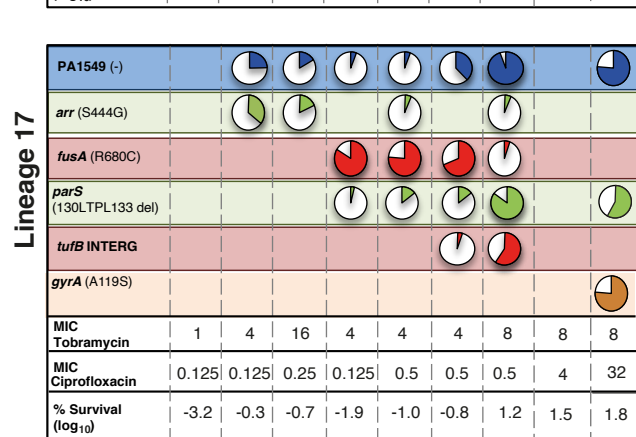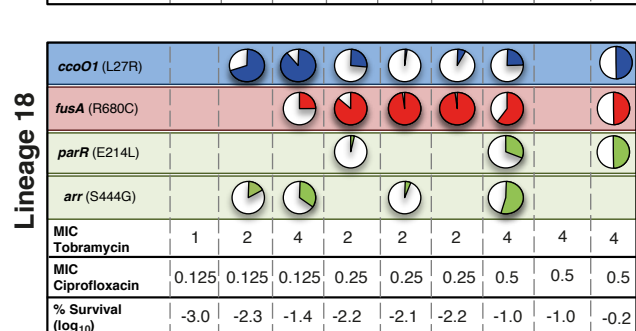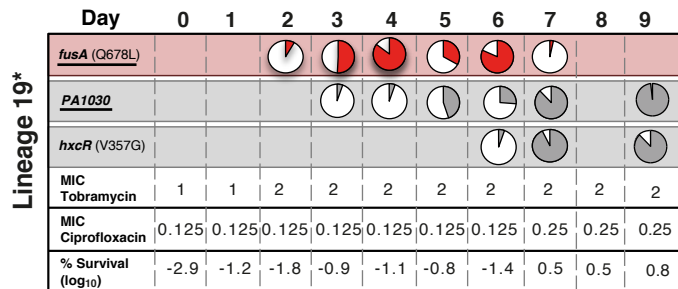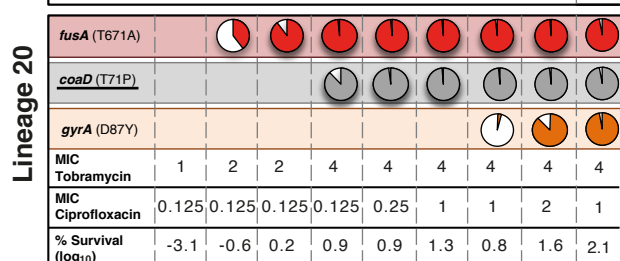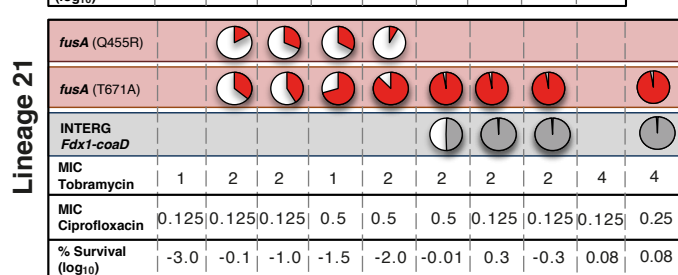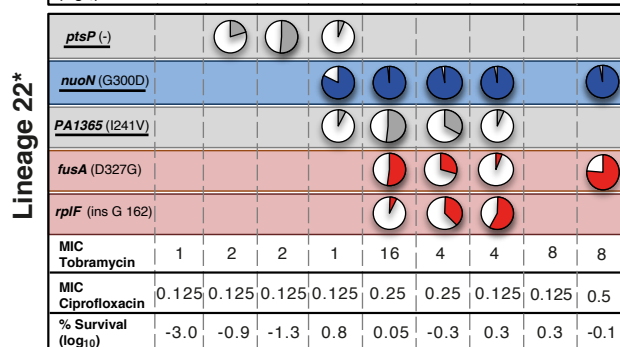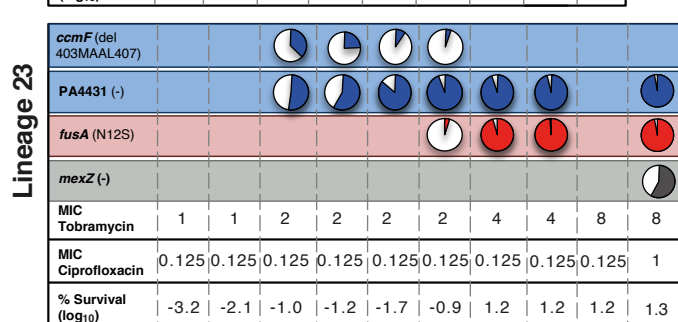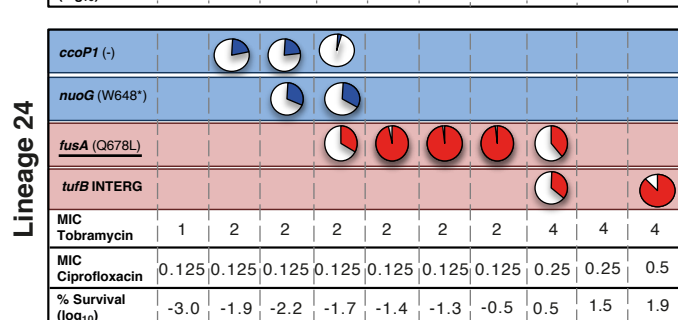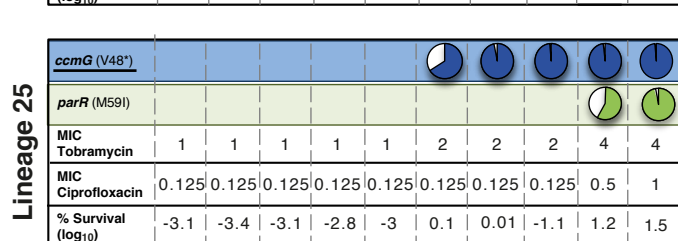

Supplement: FIG S4 [file mBio.03482-20-sf004.pdf]

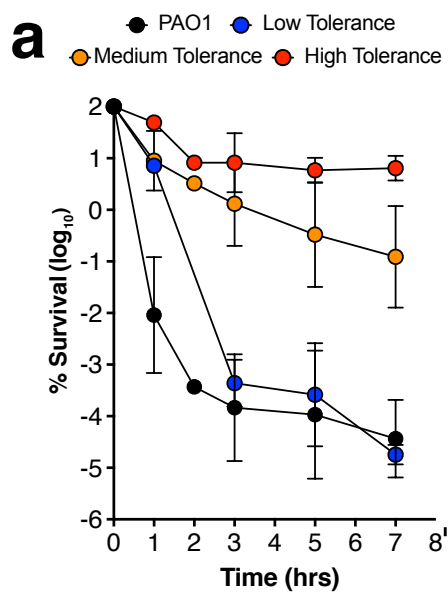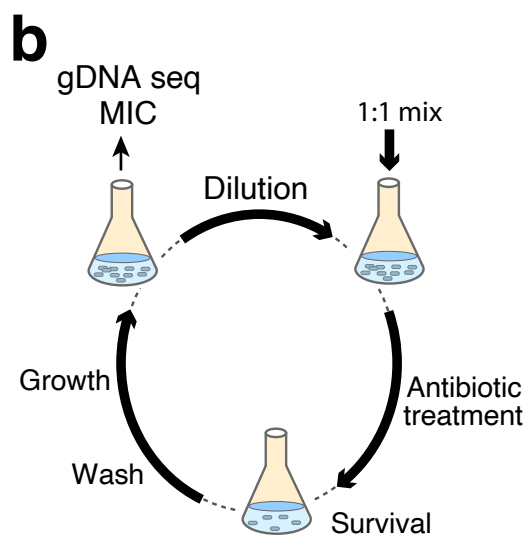

Supplement: FIG S6 [file mBio.03482-20-sf006.pdf]

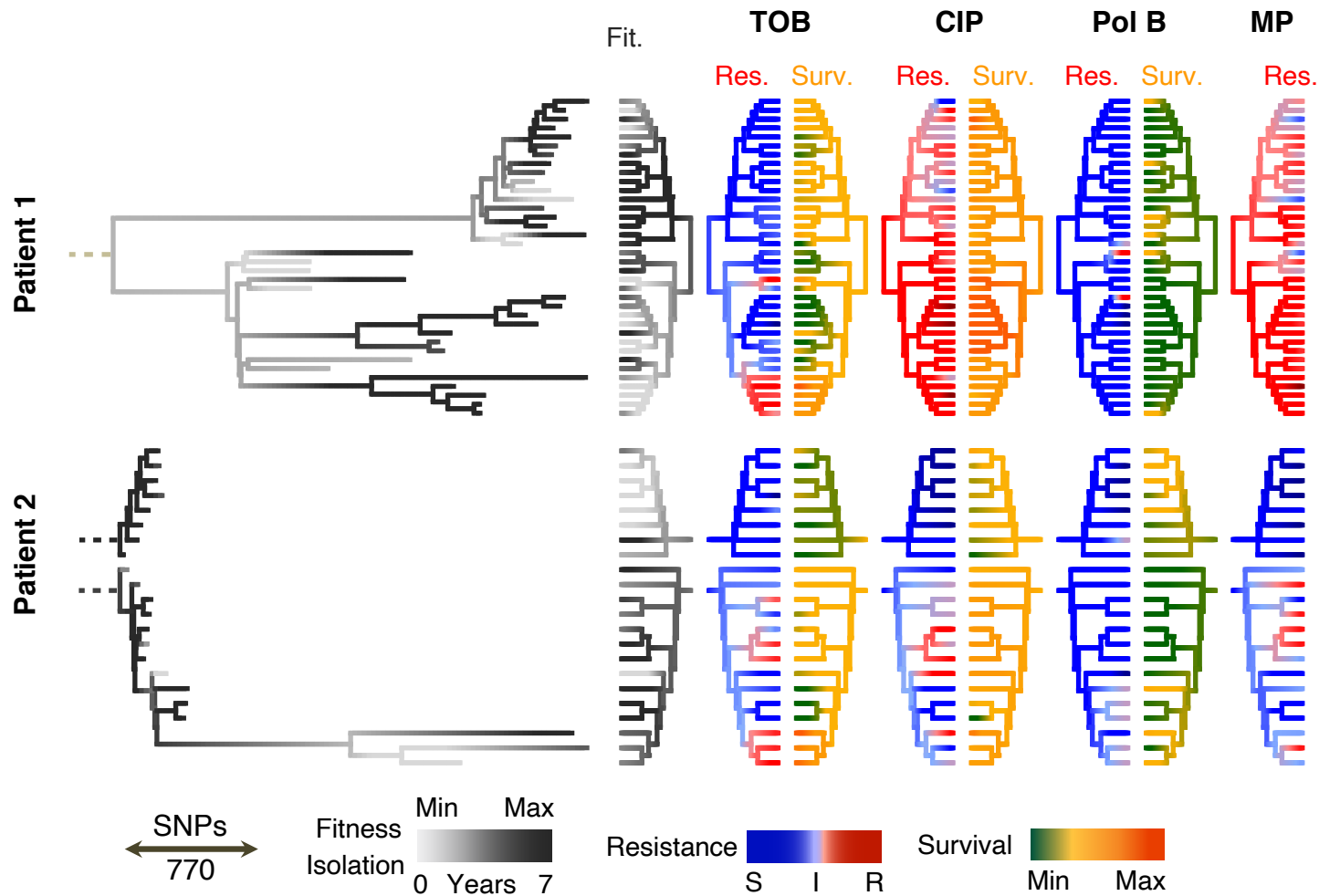

Supplement: FIG S7 [file mBio.03482-20-sf007.pdf]

## Patient 1

common to all the strains

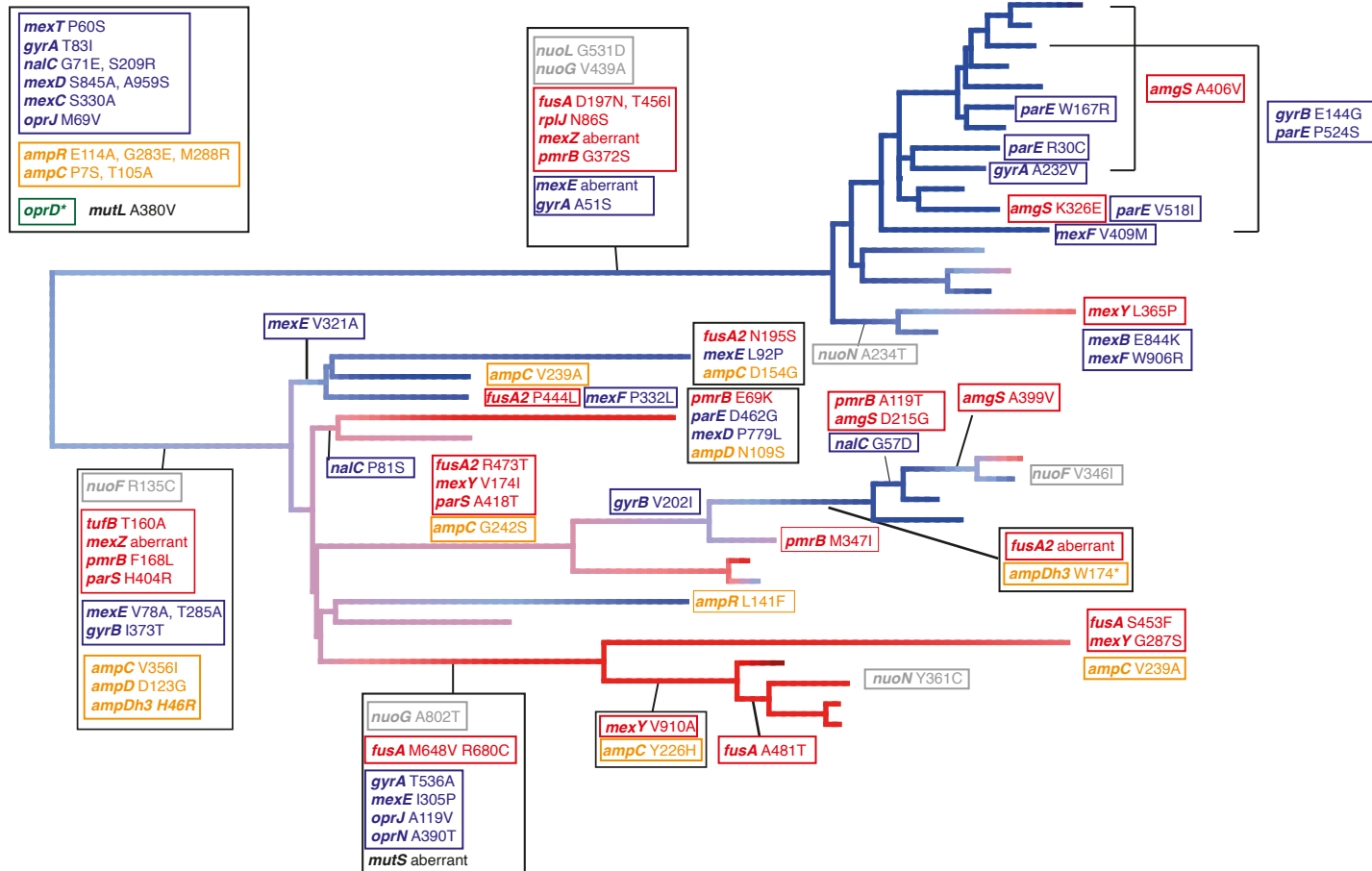

## Patient 2

common to the branch A

common to all the strains

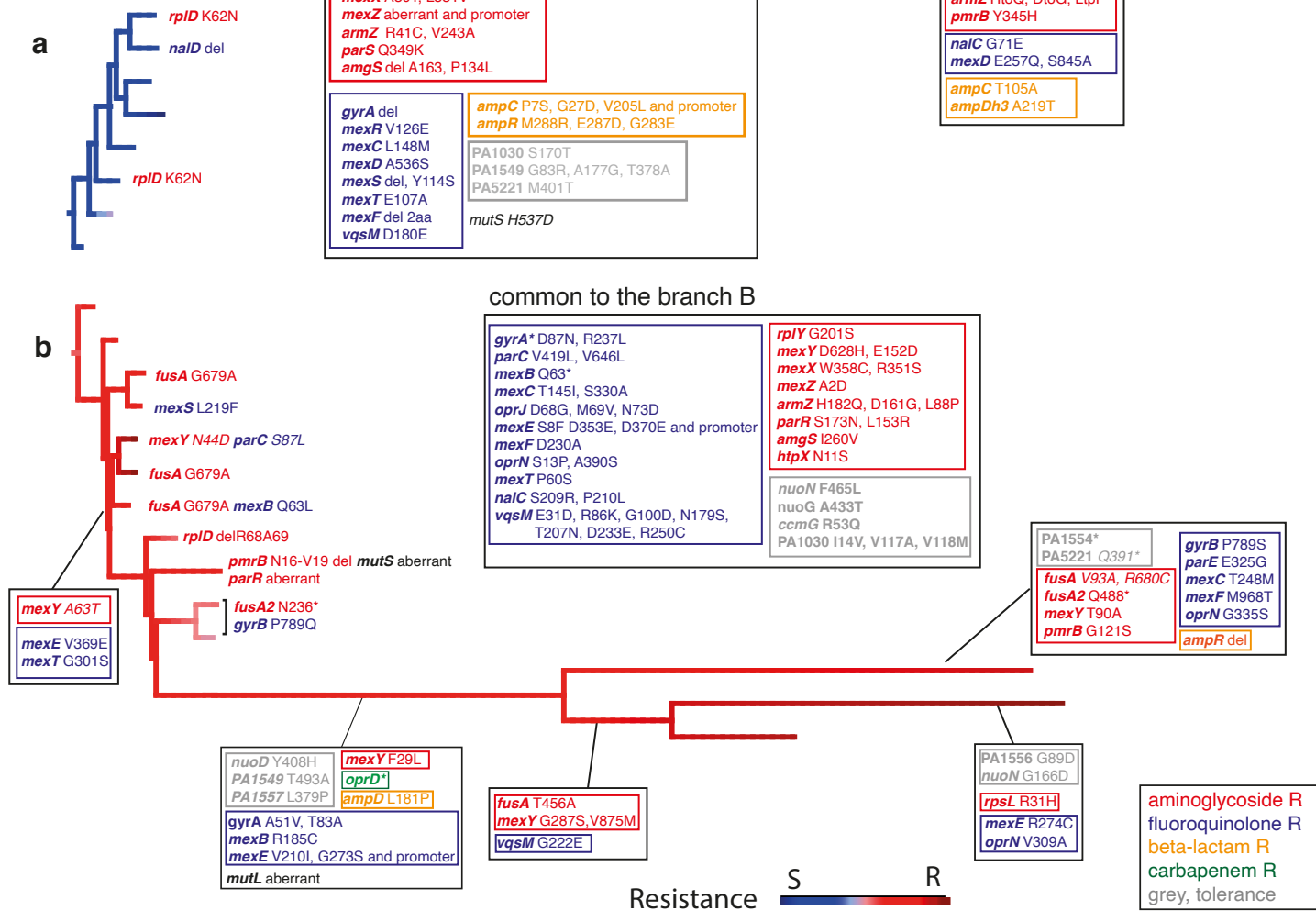

Supplement: FIG S8 [file mBio.03482-20-sf008.pdf]
