# Supplementary material for: Evolution of Antibiotic Tolerance Shapes Resistance Development in Chronic Pseudomonas aeruginosa Infections
Source: mBio. 2021 Feb 9;12(1):e03482-20. doi: 10.1128/mBio.03482-20 (PMC7885114; doi:10.1128/mBio.03482-20)
Supplement: FIG S5 [file mBio.03482-20-sf005.pdf]

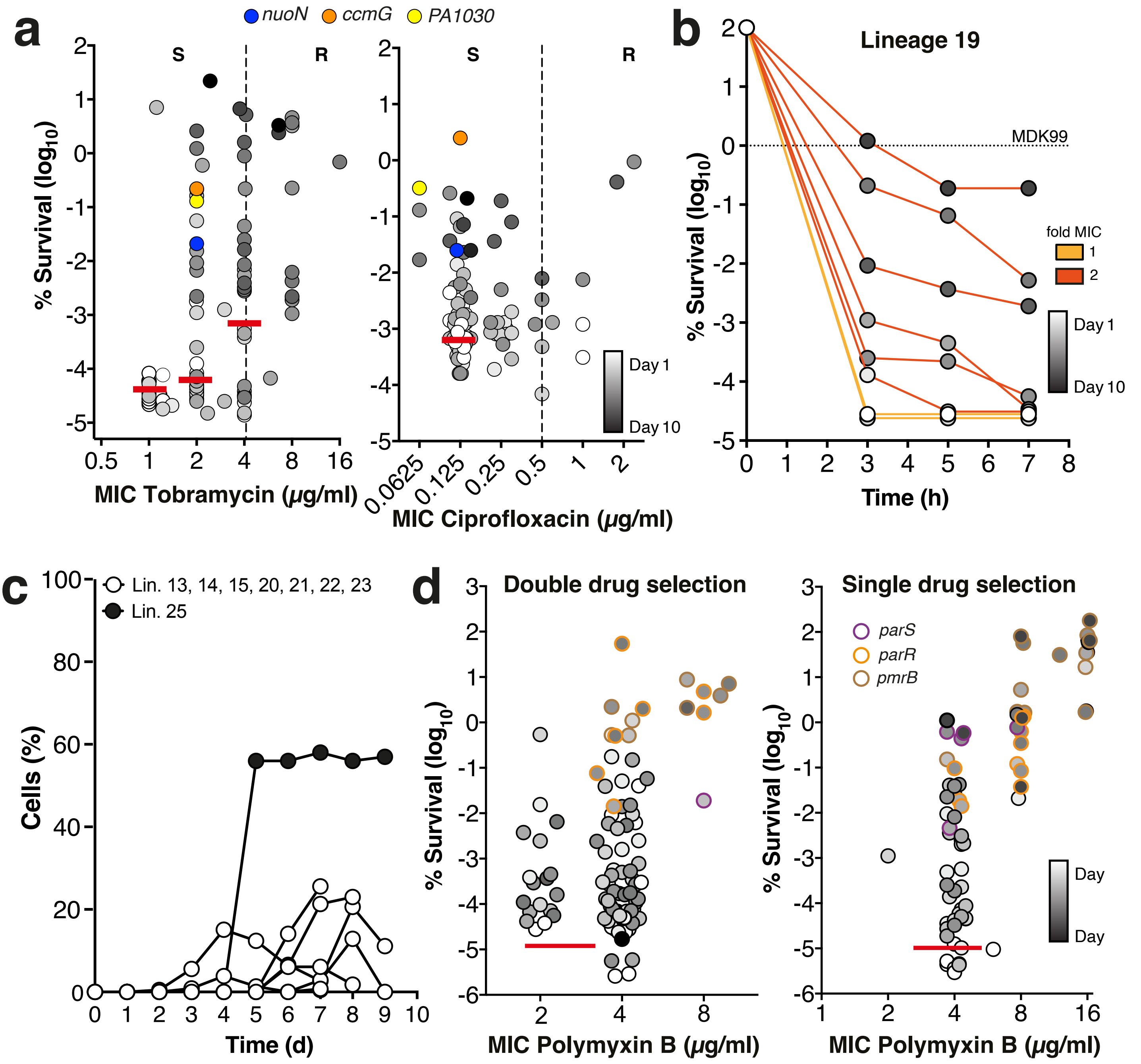

e

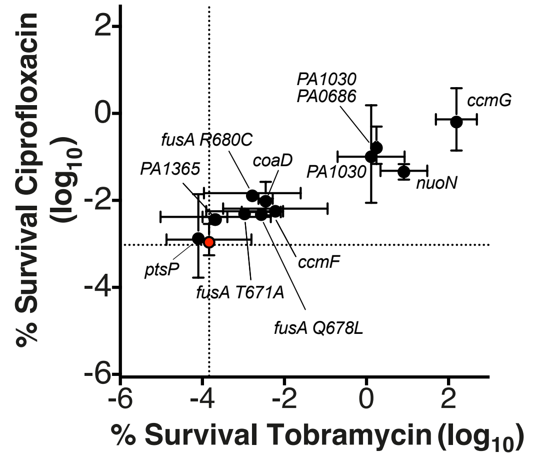

Gene	MIC (μg/ml) Tobramycin	Ciprofloxacin
ccmG	2	0.062
nuoN	2	0.125
PA1030	2	0.062
PA1030 PA0686	2	0.062
coaD	2	0.25
ccmF	2	0.125
PA1365	1	0.125
ptsP	1	0.125
fusA T671A	3	0.125
fusA Q678L	3	0.125
fusA R680C	3	0.125
