# Supplementary material for: Evolution of Antibiotic Tolerance Shapes Resistance Development in Chronic Pseudomonas aeruginosa Infections
Source: mBio. 2021 Feb 9;12(1):e03482-20. doi: 10.1128/mBio.03482-20 (PMC7885114; doi:10.1128/mBio.03482-20)
Supplement: TABLE S2 [file mBio.03482-20-st002.docx]

## Supplementary Table 2. List of strains and plasmids used in this study

| **Strains** | **Description** | | | **Reference** |
| --- | --- | --- | --- | --- |
| PAO1 | WT *P. aeruginosa* | | | (1) |
| PA14 | WT *P. aeruginosa* UCBPP-PA1 4 | | | (2) |
| *nuoN** | strain with a G300A mutation in NuoN protein | | | This study |
| *nuoD** | strain with a R551P mutation in NuoD protein | | | This study |
| *nuoM** | strain with a deletion of F184 in NuoM protein | | | This study |
| PA5221* | strain with a T233I mutation in PA5221 protein | | | This study |
| PA1549* | strain with a deletion from nucleotide 547 to nucleotide 763 resulting in aberrant protein | | | This study |
| *fusA* Y630C | strain with a Y630C mutation in FusA protein | | | This study |
| *fusA* R680C | strain with a R680C mutation in FusA protein | | | This study |
| *fusA* T671A | strain with a T671A mutation in FusA protein | | | This study |
| *fusA* Q678L | strain with a Y678C mutation in FusA protein | | | This study |
| *parS** | strain with a G388D in the ParS protein | | | This study |
| *ccmG** | strain with a stop codon at residue 49 in the CcmG protein | | | This study |
| *coaD** | strain with a T71Q mutation in the CoaD protein | | | This study |
| PA1030* | strain with a E29D mutation in the PA1030 protein | | | This study |
| PA1030*  PA0686* | strain with a E29D mutation in the PA1030 protein and V357G mutation in the PA0686 protein | | | This study |
| PA1480* | strain with a 403MAAL407 deletion in the PA1480 protein | | | This study |
| **Deletion construct** | | |  |  |
| pEX18-Tc | | *oriT*+, *sacB*+, gene replacement vector, Tc^R^ | | (3) |
| pEX18-Tc-*nuoN** | | pEX18-Tc carrying the G to A in the *nuoN* allele as *Hin*dIII-*Xba*I fragment | | This study |
| pEX18-Tc-*nuoD** | | pEX18-Tc carrying the G to C in the *nuoD* allele as *Hin*dIII-*Xba*I fragment | | This study |
| pEX18-Tc-*nuoM** | | pEX18-Tc carrying the CTT deletion *nuoM* allele as *Hin*dIII-*Xba*I fragment | | This study |
| pEX18-Tc-*fusA* Y630C | | pEX18-Tc carrying the T to C in the *fusA* allele as *Hin*dIII-*Xba*I fragment | | This study |
| pEX18-Tc-*fusA* Q678L | | pEX18-Tc carrying the T to A in the *fusA* allele as *Hin*dIII-*Xba*I fragment | | This study |
| pEX18-Tc-*PA1549** | | pEX18-Tc carrying the 216bp deletion in the *PA1549* allele as *Hin*dIII-*Xba*I fragment | | This study |
| pEX18-Tc-*PA5221** | | pEX18-Tc carrying the G to A in the *PA5221* allele as *Hin*dIII-*Xba*I fragment | | This study |
| pEX18-Tc-*ccmG** | | pEX18-Tc carrying the stop codon in the *ccmG* allele as *Hin*dIII-*Xba*I fragment | | This study |
| pEX18-Tc-*coaD** | | pEX18-Tc carrying T to G in the *coaD* allele as *Hin*dIII-*Xba*I fragment | | This study |
| pEX18-Tc-*parS** | | pEX18-Tc carrying the C to T in the *parS* allele as *Hin*dIII-*Xba*I fragment | | This study |
| pEX18-Tc-PA1030* | | pEX18-Tc carrying the G to T in the PA1030 allele as *Hin*dIII-*Xba*I fragment | | This study |

1. Holloway BW. 1955. Genetic recombination in *Pseudomonas aeruginosa*. J Gen Microbiol 13:572-81.

2. Rahme LG, Stevens EJ, Wolfort SF, Shao J, Tompkins RG, Ausubel FM. 1995. Common virulence factors for bacterial pathogenicity in plants and animals. Science 268:1899-902.

3. Hoang TT, Karkhoff-Schweizer RR, Kutchma AJ, Schweizer HP. 1998. A broad-host-range Flp-FRT recombination system for site-specific excision of chromosomally-located DNA sequences: application for isolation of unmarked *Pseudomonas aeruginosa* mutants. Gene 212:77-86.
