# Supplementary material for: Evolution of Antibiotic Tolerance Shapes Resistance Development in Chronic Pseudomonas aeruginosa Infections
Source: mBio. 2021 Feb 9;12(1):e03482-20. doi: 10.1128/mBio.03482-20 (PMC7885114; doi:10.1128/mBio.03482-20)
Supplement: TABLE S3 [file mBio.03482-20-st003.docx]

## Supplementary Table 3. Oligos used in this study

| **Primers** | **Description** | **Sequence** |
| --- | --- | --- |
| A | nuoN*_F | gat**AAGCTT**CCTGTTCATCGGCCTGGAGCTGCTC |
| B | nuoN*_R | gcg**TCTAGA**TCAGCCGGCGAGGGCCAGGCCGGAAT |
| C | nuoD*_F | gat**AAGCTT**TGGTCCGCGAGTTCCTCGACTGGATG |
| D | nuoD*_R | gcg**TCTAGA**TGGGTGTCGTCGTCGATCATCAGCGC |
| E | nuoM*_F | gat**AAGCTT**TGTACGACAAGCTGTTCGTGAAA |
| F | nuoM*_R | gcg**TCTAGA**GAGTAGATGCCGATCATCACGAA |
| G | fusA*_F | gat**AAGCTT**TCAACCTTGTTTTTTAACCAGCGCT |
| H | fusA*_R | gcg**TCTAGA**CGCAGGCGACATCGCTGCCCTGATC |
| I | PA1549*_F | gat**AAGCTT**ATGAGCGCCCCCCTGCCCTGCTACCAC |
| J | PA1549*_R | gcg**TCTAGA**GCTGGTGGTCGACCTGCCACCACAGCG |
| K | ccmG*_F | gat**AAGCTT**TTCGATGTACTTCGGCGATTTCAA |
| L | ccmG*_R | gcg**TCTAGA**TTGTAGCGAACGAAATCACCGTA |
| M | coaD*_F | gcg**AAGCTT**ACTCGGTGCAGAGATTGGGATC |
| N | coaD*_R | gcg**TCTAGAA**CCACCTGCGGAACCTGTCGAT |
| O | parS*_F | gat**AAGCTT**ACGTCCTGCCTTTCCATTTCCC |
| P | parS*_R | gcg**TCTAGA**TTCTACGACAGCATCGTCGAGAA |
| Q | PA5221*_F | gat**AAGCTT**GTCCGAAGCCAGCCGGCGGATTCTCC |
| R | PA5221*_R | gcg**TCTAGA**TCAGACTCGTGCCAGCATCGGCAGGT |
| S | PA1030*_F | gat**AAGCTT**GAGCGCTCTCATCAAGGAACGTCCC |
| T | PA1030*_R | gcg**TCTAGA**CTTTCCGTCCACCAGGAACAGCTCC |
