# Supplementary material for: Evolution of Antibiotic Tolerance Shapes Resistance Development in Chronic Pseudomonas aeruginosa Infections
Source: mBio. 2021 Feb 9;12(1):e03482-20. doi: 10.1128/mBio.03482-20 (PMC7885114; doi:10.1128/mBio.03482-20)
Supplement: TABLE S4 [file mBio.03482-20-st004.docx]

## Supplementary Table 4. Genome sequences of clinical isolates

| **strain** | **BioSample** | **SRA** | **NCBI_Accession** |
| --- | --- | --- | --- |
| UJPc172 | SAMN12501671 | SRS5215544 | 12501671 |
| UJPc173 | SAMN12501670 | SRS5215543 | 12501670 |
| UJPc174 | SAMN12501648 | SRS5215545 | 12501648 |
| UJPc175 | SAMN12501647 | SRS5215550 | 12501647 |
| UJPc176 | SAMN12501646 | SRS5215551 | 12501646 |
| UJPc237 | SAMN12501644 | SRS5215536 | 12501644 |
| UJPc238 | SAMN12501643 | SRS5215535 | 12501643 |
| UJPc239 | SAMN12501642 | SRS5215534 | 12501642 |
| UJPc266 | SAMN12501640 | SRS5215541 | 12501640 |
| UJPc267 | SAMN12501639 | SRS5215539 | 12501639 |
| UJPc268 | SAMN12501638 | SRS5215538 | 12501638 |
| UJPc327 | SAMN12501669 | SRS5215533 | 12501669 |
| UJPc328 | SAMN12501668 | SRS5215531 | 12501668 |
| UJPc399 | SAMN12501637 | SRS5215563 | 12501637 |
| UJPc400 | SAMN12501636 | SRS5215562 | 12501636 |
| UJPc401 | SAMN12501635 | SRS5215568 | 12501635 |
| UJPc402 | SAMN12501634 | SRS5215569 | 12501634 |
| UJPc403 | SAMN12501633 | SRS5215566 | 12501633 |
| UJPc404 | SAMN12501632 | SRS5215567 | 12501632 |
| UJPc418 | SAMN12501667 | SRS5215572 | 12501667 |
| UJPc419 | SAMN12501666 | SRS5215571 | 12501666 |
| UJPc449 | SAMN12501631 | SRS5215552 | 12501631 |
| UJPc450 | SAMN12501630 | SRS5215553 | 12501630 |
| UJPc451 | SAMN12501629 | SRS5215556 | 12501629 |
| UJPc452 | SAMN12501628 | SRS5215554 | 12501628 |
| UJPc453 | SAMN12501627 | SRS5215557 | 12501627 |
| UJPc454 | SAMN12501626 | SRS5215555 | 12501626 |
| UJPc483 | SAMN12501665 | SRS5215559 | 12501665 |
| UJPc484 | SAMN12501664 | SRS5215558 | 12501664 |
| UJPc529 | SAMN12501625 | SRS5215560 | 12501625 |
| UJPc530 | SAMN12501624 | SRS5215589 | 12501624 |
| UJPc531 | SAMN12501623 | SRS5215530 | 12501623 |
| UJPc532 | SAMN12501622 | SRS5215532 | 12501622 |
| UJPc550 | SAMN12501621 | SRS5215598 | 12501621 |
| UJPc551 | SAMN12501620 | SRS5215599 | 12501620 |
| UJPc552 | SAMN12501619 | SRS5215600 | 12501619 |
| UJPc553 | SAMN12501618 | SRS5215601 | 12501618 |
| UJPc570 | SAMN12501617 | SRS5215585 | 12501617 |
| UJPc572 | SAMN12501615 | SRS5215583 | 12501615 |
| UJPc573 | SAMN12501614 | SRS5215582 | 12501614 |
| UJPc588 | SAMN12501663 | SRS5215581 | 12501663 |
| UJPc589 | SAMN12501662 | SRS5215580 | 12501662 |
| UJPc590 | SAMN12501661 | SRS5215579 | 12501661 |
| UJPc607 | SAMN12501613 | SRS5215578 | 12501613 |
| UJPc608 | SAMN12501612 | SRS5215577 | 12501612 |
| UJPc609 | SAMN12501611 | SRS5215576 | 12501611 |
| UJPc705 | SAMN12501660 | SRS5215525 | 12501660 |
| UJPc724 | SAMN12501659 | SRS5215522 | 12501659 |
| UJPc725 | SAMN12501658 | SRS5215523 | 12501658 |
| UJPc755 | SAMN12501657 | SRS5215528 | 12501657 |
| UJPc774 | SAMN12501656 | SRS5215609 | 12501656 |
| UJPc775 | SAMN12501655 | SRS5215607 | 12501655 |
| UJPc776 | SAMN12501654 | SRS5215603 | 12501654 |
| UJPc796 | SAMN12501653 | SRS5215604 | 12501653 |
| UJPc810 | SAMN12501652 | SRS5215573 | 12501652 |
| UJPc811 | SAMN12501651 | SRS5215570 | 12501651 |
| UJPc812 | SAMN12501650 | SRS5215595 | 12501650 |
| UJPc813 | SAMN12501649 | SRS5215597 | 12501649 |
